# A pharynx-to-brain axis controls pharyngeal inflammation–induced anxiety

**DOI:** 10.1073/pnas.2312136121

**Published:** 2024-03-06

**Authors:** Wan Zhao, Ke Zhang, Wan-Ying Dong, Hao-Di Tang, Jia-Qiang Sun, Ji-Ye Huang, Guang-Lun Wan, Rui-Rui Guan, Xiao-Tao Guo, Ping-Kai Cheng, Ran Tao, Jing-Wu Sun, Zhi Zhang, Xia Zhu

**Affiliations:** ^a^Department of Otolaryngology-Head and Neck Surgery, The First Affiliated Hospital of University of Science and Technology of China, Division of Life Sciences and Medicine, University of Science and Technology of China, Hefei 230001, People’s Republic of China; ^b^Department of Neurobiology and Biophysics, Hefei National Laboratory for Physical Sciences at the Microscale, Division of Life Sciences and Medicine, University of Science and Technology of China, Hefei 230026, People’s Republic of China; ^c^Laboratory of Anesthesia and Critical Care Medicine Department of Anesthesia and Critical Care Laboratory, National-Local Joint Engineering Research Center of Translational Medicine of Anesthesiology, West China Hospital, Sichuan University, Chengdu 610041, People’s Republic of China; ^d^Department of Vascular Surgery, The Second Hospital of Anhui Medical University, Hefei 230601, People’s Republic of China; ^e^The Center for Advanced Interdisciplinary Science and Biomedicine, Institute of Health and Medicine, Division of Life Sciences and Medicine, University of Science and Technology of China, Hefei 230026, People’s Republic of China

**Keywords:** pharyngeal inflammation–induced anxiety, brain–body interactions, neural circuits, quadruple trans-monosynaptic tracing, in vivo calcium imaging

## Abstract

Anxiety is a remarkably common condition among patients with pharyngitis, but the relationship between these disorders has received little research attention, and the underlying neural mechanisms remain unknown. Here, we show that the densely innervated pharynx transmits signals induced by pharyngeal inflammation to glossopharyngeal and vagal sensory neurons of the nodose/jugular/petrosal (NJP) superganglia in mice. Specifically, the NJP superganglia project to norepinephrinergic neurons in the nucleus of the solitary tract (NTS^NE^). These NTS^NE^ neurons project to the ventral bed nucleus of the stria terminalis (vBNST) that induces anxiety-like behaviors in a murine model of pharyngeal inflammation. Inhibiting this pharynx→NJP→NTS^NE^→vBNST circuit can alleviate anxiety-like behaviors associated with pharyngeal inflammation. This study thus defines a pharynx-to-brain axis that mechanistically links pharyngeal inflammation and emotional response.

## Significance

Anxiety is an extremely common phenomenon among outpatients with pharyngitis, the latter of which is difficult to treat in the clinic. The relationship between anxiety and pharyngitis, as well as the neural circuit mechanisms potentially responsible for their comorbidity, have been all but ignored in research. Using extensive state-of-the-art tools and methods for neuroscience research, we identify a pharynx-to-brain axis that primes pharyngeal inflammation–induced anxiety. This work defining a pharynx-to-brain axis responsible for linking pharyngeal inflammation with anxiety via glossopharyngeal and vagal nerves holds major implications for expanding our understanding the connection between inflammation, sensory stimuli, and emotional response.

Pharyngitis manifests as inflammation of the pharyngeal mucous membrane (1). At least 20% of the world’s population suffers from pharyngitis, making it one of the most common reasons for a visit to a primary care physician (2, 3). Anxiety is commonly reported among patients with pharyngitis admitted to outpatient clinics (4), but the underlying neural mechanisms remain largely unknown.

Pharyngeal inflammation may be caused by infectious factors (e.g., typically viruses or bacteria), physiological disorders (e.g., exposure to gastric pepsin due to laryngopharyngeal reflux), unhealthy lifestyle (e.g., smoking or sleep apnea), or environmental conditions (e.g., air contaminants) (5, 6). How the brain senses the pharyngeal inflammation to produce emotional responses is unclear. The pharynx is densely innervated by the glossopharyngeal and vagal nerves (7–9). Previous murine studies using cholera toxin or wheat germ agglutinin conjugated horseradish peroxidase neural tracers discovered that glossopharyngeal and vagal sensory fibers in the pharynx arise from the pseudo-unipolar nodose/jugular/petrosal (NJP) sensory neurons, which then predominantly project to the nucleus of the solitary tract (NTS) (10, 11). However, limitations in the tracing tools and strategies for targeted manipulation of ganglia-projecting neurons have prevented a clear understanding of the precise cell type–specific organization and function(s) of pathways between the pharynx and brain in pharyngitis-associated anxiety.

In the present study, we investigated the cell type–specific organization and functions of a pharynx-to-brain axis necessary for pharyngeal inflammation–induced anxiety. Through viral tracing and chemogenetic manipulation, we found that inflammatory stimuli in the pharynx are transmitted by pharynx-projecting glutamatergic sensory neurons in the NJP to norepinephrinergic neurons in the nucleus of the solitary tract (NTS^NE^), and inhibiting this pathway can alleviate anxiety-like behaviors in the murine model of pharyngeal inflammation. In turn, NTS^NE^ neurons innervate the ventral bed nucleus of the stria terminalis (vBNST). Through wireless optogenetic manipulation, in vivo microendoscopic calcium imaging, and multitetrode electrophysiological recordings in freely moving mice, we found that inhibition of NTS^NE^ inputs to the vBNST also reduce pharyngeal inflammation–associated anxiety-like behaviors in mice. Through multiple lines of evidence, this study defines a pharynx-to-brain axis via glossopharyngeal and vagal nerves that is required for the induction of anxiety associated with pharyngeal inflammation, suggesting multiple potential therapeutic targets for relieving anxiety and mood disorders in patients with pharyngitis.

## Results

### Pharyngeal Inflammation Is Associated with Anxiety in Humans and Mice.

**Fig. 1.**
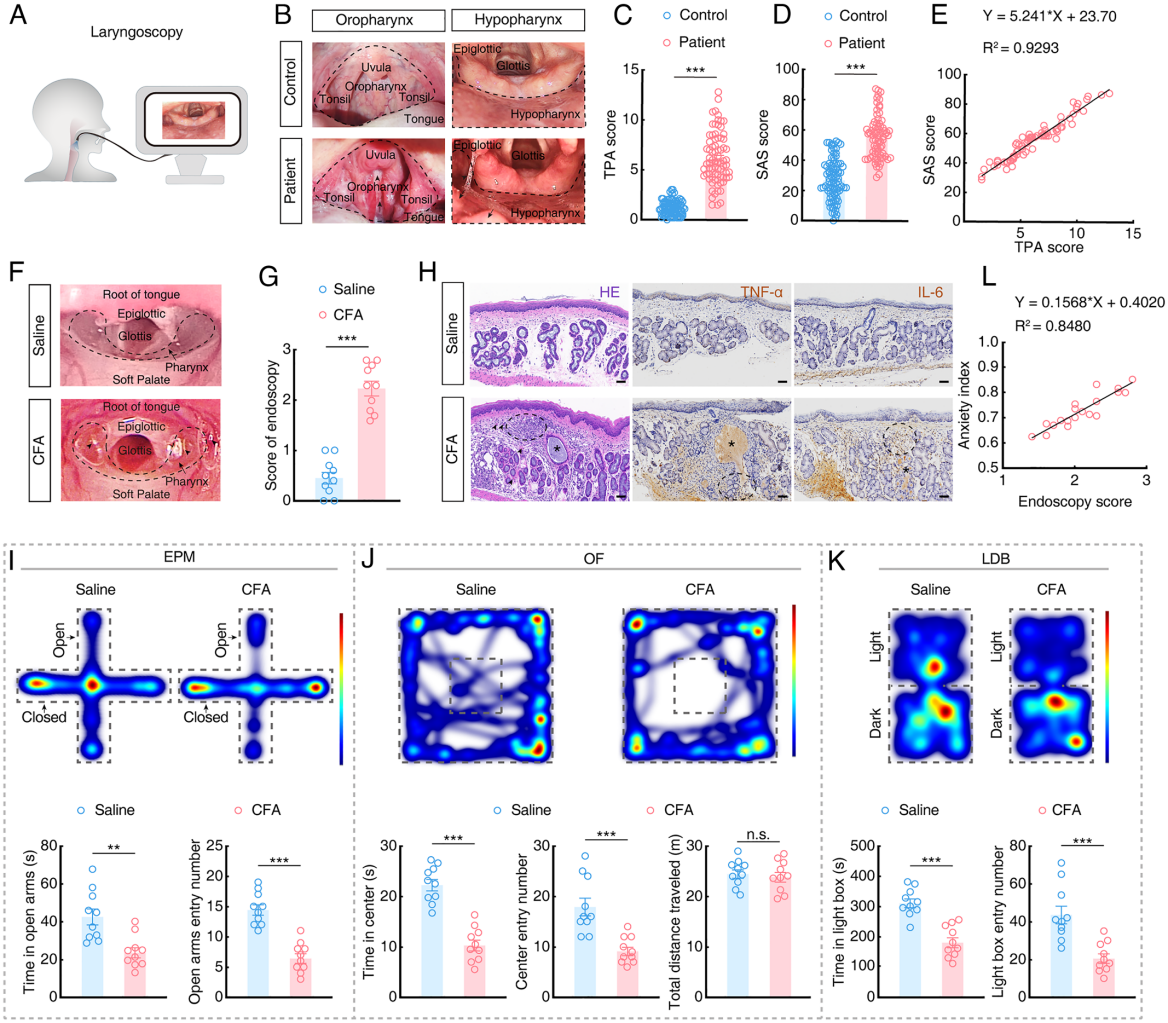
Pharyngeal inflammation is associated with anxiety in humans and mice. (*A*) Schematic of the laryngoscopy of enrolled subjects. (*B*) Representative laryngoscopic images showing the oropharynx and hypopharynx in healthy controls and patients with pharyngitis. The dashed box shows the oropharynx and hypopharynx of subjects, which are more congested in patients with pharyngitis than those in the healthy group; the long arrow (➛) indicates hyperplastic lymphoid follicles, and the short arrow (➤) indicates secretions. (*C*) The Tonsillo-Pharyngitis Assessment (TPA) score in healthy controls and patients with pharyngitis (control, *n* = 86; patient, *n* = 76; *t*_160_ = 17.42, *P* < 0.0001). (*D*) The Self-Rating Anxiety Scale (SAS) score in healthy controls and patients with pharyngitis (control, *n* = 86; patient, *n* = 76; *t*_160_ = 13.11, *P* < 0.0001). (*E*) Correlation between the TPA score and SAS score of patients (*n* = 76, Pearson’s: *R*^2^ = 0.9293, *P* < 0.0001). (*F*) Representative pharyngeal endoscopic images of saline and CFA mice. The dashed box and the long arrow (➛) indicate the pharynx of mice, which is more congested in CFA mice than in the saline group, with the short arrow (➤) indicating secretions. (*G*) The endoscopy score for saline and CFA mice (*n* = 10 mice per group; *t*_18_ = 9.637, *P* < 0.0001). (*H*) Representative images showing hematoxylin-eosin (H&E), tumor necrosis factor-α (TNF-α, inflammatory cytokine), and interleukin-6 (IL-6, inflammatory cytokine) staining of the pharyngeal mucosa of saline and CFA mice. The short arrow (➤) indicates hemorrhagic spots, and the asterisk (*) indicates retention of secretions in the mucous glands, and the dashed box indicates high infiltration of inflammatory cells or factors. (Scale bars, 50 µm.) (*I*) Representative heatmaps of the travel trajectory of saline and CFA mice and summarized data for the time spent in and the number of entering the open arms in the elevated plus maze (EPM) test (*n* = 10 mice per group; *Left*, *t*_18_ = 3.798, *P* = 0.0013; *Right*, *t*_18_ = 6.905, *P* < 0.0001). (*J*) Representative heatmaps of the travel trajectory and summarized data for the time spent in and the number of entering the center of the open field (OF) and the total distance traveled by saline and CFA mice in the OF test (*n* = 10 mice per group; *Left*, *t*_18_ = 7.646, *P* < 0.0001; *Middle*, *t*_18_ = 4.495, *P* = 0.0003; *Right*, *t*_18_ = 0.4801, *P* = 0.6369). (*K*) Representative heatmaps of the travel trajectory of saline and CFA mice and summarized data for the time spent in and the number of entering the light box in the light–dark box (LDB) test (*n* = 10 mice per group; *Left*, *t*_18_ = 5.951, *P* < 0.0001; *Right*, *t*_18_ = 4.373, *P* = 0.0004). (*L*) Correlation between the endoscopy score and anxiety index of mice with injected different doses (5 μL, 10 μL) of CFA (*n* = 20, Pearson’s: *R*^2^ = 0.8480, *P* < 0.0001). Significance was assessed by two-tailed unpaired Student’s *t* tests in (*C*, *D*, *G*, and *I–K*), and Pearson’s correlations in (*E* and *L*). All data are presented as the mean ± SEM. **P < 0.01; ***P < 0.001, n.s., not significant.

To assess the correlation between pharyngeal inflammation and anxiety, we enrolled subjects with pharyngitis, together with healthy volunteers. Laryngoscopic imaging showed that patients with pharyngitis presented with more obvious congestion, increased mucous secretion, and lymphoid follicular hyperplasia in oropharyngeal and hypopharyngeal areas compared to healthy volunteers (Fig. 1 *A* and *B*). We then determined a Tonsillo-Pharyngitis Assessment (TPA) to assess the severity of pharyngitis (12). We found that patients with pharyngitis had higher TPA scores than control subjects (Fig. 1*C*). In addition, we evaluated the severity of anxiety in this cohort using a Self-rating Anxiety Scale (SAS) (13), and found that patients with pharyngitis had higher SAS scores than healthy controls (Fig. 1*D*). Furthermore, a Pearson correlation analysis indicated that TPA scores were strongly positively correlated with SAS scores in patients with pharyngitis (Fig. 1*E*). These data suggested that pharyngitis is commonly accompanied with anxiety in patients.

In order to investigate the precise mechanistic relationship between pharyngitis and anxiety, we developed a murine model of pharyngeal inflammation by injection of Complete Freund’s Adjuvant (CFA, *SI Appendix*, Fig. S1*A*), a classical inflammatory factor consisting of heat-killed *Mycobacterium tuberculosis* in nonmetabolizable oils (paraffin oil and mannide monooleate) into pharynx (14–16). To this end, at 7 d after pharynx injection, endoscopic observation showed that mucosal congestion, edema, and secretions were obviously higher in the CFA mice (5 µL or 10 µL) (Fig. 1 *F* and *G* and *SI Appendix*, Fig. S1 *B* and *C*), accompanying with increased occurrence of hypertrophy, hemorrhagic spots, inflammatory cell infiltration and proinflammatory cytokines by hematoxylin-eosin staining, compared with saline control mice (Fig. 1*H*) (17). Behavioral tests, including the elevated plus maze (EPM), the open field (OF), and the light–dark box (LDB), showed that CFA mice (5 µL or 10 µL) exhibited significant anxiety-like behaviors with fewer entries and less time in open arms or center zone of EPM and OF, respectively, as well as fewer entries and less time in lighted area of LDB compared to saline control mice (Fig. 1 *I–K* and *SI Appendix*, Fig. S1 *D–F*). Moreover, Pearson correlation analysis identified a positive correlation between anxiety severity index values and endoscopy scores for pharyngeal inflammation (Fig. 1*L*) (18, 19). We focused on CFA mice (10 µL) throughout the study unless otherwise stated. Additionally, we observed no difference in locomotion or anorexia at 7 d after CFA injection (Fig. 1*J* and *SI Appendix*, Fig. S2).

Previous studies reported that pharyngeal inflammation could be relieved by treatment with the classic steroidal anti-inflammatory drug, dexamethasone (DXM) (20, 21). To investigate whether DXM could also relieve CFA-induced pharyngeal inflammation, DXM was intraperitoneally injected (2 mg/kg) daily for 7 d beginning at the day of pharyngeal injection with CFA (*SI Appendix*, Fig. S3*A*). We found that DXM attenuated pharyngeal inflammation and anxiety-like behaviors in CFA mice (*SI Appendix*, Fig. S3 *B*–*F*). These results suggested that pharyngeal inflammation was associated with anxiety in humans and mice.

### Defining Functional Connections of the Pharynx→NJP→NTS^NE^ Circuit.

**Fig. 2.**
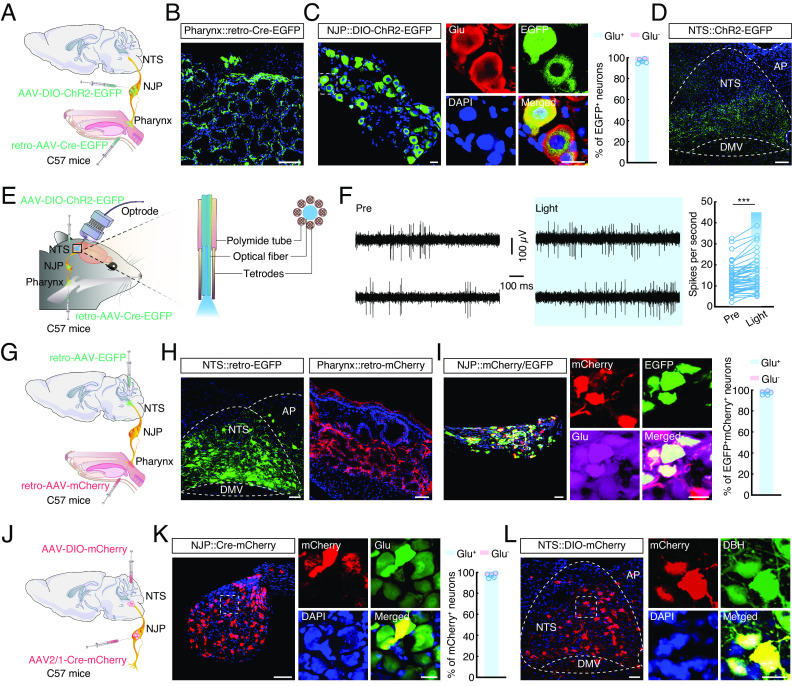
Defining functional connections of the pharynx→NJP→NTS^NE^ circuit. (*A*) Schematic of the viral injection. (*B*) Representative image showing the infusion site within the pharynx. (Scale bar, 100 µm.) (*C*) Representative images showing the EGFP^+^ neurons in the nodose/jugular/petrosal (NJP) superganglia and ~99% of EGFP^+^ neurons colocalized with glutamate antibody signal. (Scale bars, 20 µm.) (*D*) Representative image of EGFP^+^ fiber expression in the nucleus of the solitary tract (NTS). (Scale bar, 100 µm.) (*E*) Schematic of viral injection and multitetrode recording in freely moving mice. (*F*) Example recordings of spontaneous and light-evoked spikes (*Left*) and summarized data recorded (*Right*) from the neurons in the NTS before and during light photostimulation of the NJP fibers (493 nm light; *n* = 46 cells; *t*_45_ = 7.252, *P* < 0.0001). (*G*) Schematic of the viral injection. (*H*) Typical images showing the infusion site within the NTS (*Left*) and pharynx (*Right*). (Scale bars, 50 μm [*Left*] or 100 μm [*Right*].) (*I*) Representative confocal images show EGFP^+^ from the NTS being detected by mCherry^+^ neurons innervating the pharynx (*Left*) and ~99% of EGFP^+^mCherry^+^ neurons in the NJP colocalized with glutamate antibody signal (*Right*). (Scale bars, 50 μm [*Left*] or 20 μm [*Right*].) (*J*) Schematic of the viral injection. (*K*) Representative images showing the infusion site within the NJP neurons (*Left*) and ~99% of mCherry^+^ neurons colocalized with glutamate antibody signal (*Right*). (Scale bars, 100 μm [*Left*] or 20 μm [*Right*].) (*L*) Representative images of mCherry^+^ neurons expressed in the NTS (*Left*) and predominantly colocalized with dopamine β-hydroxylase (DBH) antibody (*Right*). (Scale bars, 50 µm [*Left*] or 20 µm [*Right*].) Significance was assessed by two-tailed paired Student’s *t* tests in (*F*). All data are presented as the mean ± SEM. ***P < 0.001.

In mice, densely distributed glossopharyngeal and vagal nerves innervate the pharynx (22), with their afferent soma fusing to form a pair of nodose/jugular/petrosal (NJP) superganglia (23). We therefore sought to determine NJP neurons connecting the pharynx and brain that might participate in pharyngeal inflammation–induced anxiety-like behaviors. For this purpose, we combined anterograde and retrograde viral tracing methods by injecting retro-AAV-Cre-EGFP into the pharynx and an AAV expressing *Cre*-dependent channelrhodopsin-2 (AAV-DIO-ChR2-EGFP) into the NJP superganglia of C57 mice (Fig. 2 *A–C*). At 3 wk after injection, we found ~99% of the EGFP-projecting neurons in the NJP superganglia colocalized with the signal for glutamatergic antibody and EGFP^+^ fibers in the nucleus of the solitary tract (NTS) (Fig. 2 *C* and *D*), which is known to play a key role in communication between the pharynx and brain (24).

To further characterize the functional connections of this pharynx→NJP→NTS pathway, we conducted in vivo multitetrode electrophysiological recordings in the NTS of freely moving mice (Fig. 2*E*). Upon blue light (473 nm) stimulation of pharynx-projecting NJP neuronal terminals in the NTS, spike number significantly increased in the NTS neurons (Fig. 2*F*). Further retrograde tracing by injection of retro-AAV-hSyn-mCherry into the pharynx and retro-AAV-hSyn-EGFP injection into the NTS showed that mCherry^+^ EGFP^+^ colabeled neurons in the NJP superganglia (Fig. 2 *G* and *H*), ~99% of which also colocalized with the signal for glutamatergic antibody at 3 wk after injection (Fig. 2*I*). These results thus demonstrated that the pharynx was connected to the NTS through NJP superganglia.

To determine the type of neurons in the NTS that are innervated by NJP neurons, we injected AAV2/1-Cre-mCherry into the NJP superganglia for monosynaptic anterograde tracing of *Cre* and mCherry expression (25, 26), and AAV-DIO-mCherry into the NTS of C57 mice (Fig. 2*J* and *SI Appendix*, Fig. S4*A*). Three weeks later, mCherry^+^ neurons could be detected in the NJP superganglia, ~99% of which colocalized with the signal for glutamatergic antibody (Fig. 2*K*). Additionally, mCherry^+^ neurons could be also detected in the NTS (Fig. 2*L*). Immunofluorescence staining revealed that the large majority of mCherry^+^ neurons colocalized with antibody signal for dopamine β-hydroxylase (DBH, which is the marker of norepinephrine (NE) neuron, ~78%) (Fig. 2*L* and *SI Appendix*, Fig. S4 *B* and *C*), and to a lesser extent with antibody signal for γ-aminobutyric acid (GABA, ~5%) (*SI Appendix*, Fig. S4 *B* and *D*), 5-hydroxytryptamine (5-HT, ~4%) (*SI Appendix*, Fig. S4 *B* and *E*) and neuron-derived neuropeptide Y (NPY, ~3%) (*SI Appendix*, Fig. S4 *B* and *F*). Together, our results demonstrate a pharynx→NJP→NTS^NE^ circuit.

### The Pharynx→NJP→NTS^NE^ Circuit Controls Pharyngeal Inflammation–Induced Anxiety.

To investigate whether NJP neuronal activity is affected by pharyngeal inflammation, we conducted immunofluorescence staining for c-Fos in NJP neurons. We found that c-Fos levels were increased in the NJP neurons at 7 d after CFA injection compared to that in controls (*SI Appendix*, Fig. S5 *A* and *B*). To determine whether this increase in c-Fos levels was mediated by glossopharyngeal and vagal nerves, these nerves were transected prior to CFA injection. At 7 d after injection, the CFA-induced increase in c-Fos levels in the NJP neurons was largely eliminated (*SI Appendix*, Fig. S5*C*). Moreover, in CFA mice without nerve transection, ~99% of c-Fos^+^ neurons in NJP neurons colocalized with the signal for glutamatergic antibody (*SI Appendix*, Fig. S5 *D* and *E*).

**Fig. 3.**
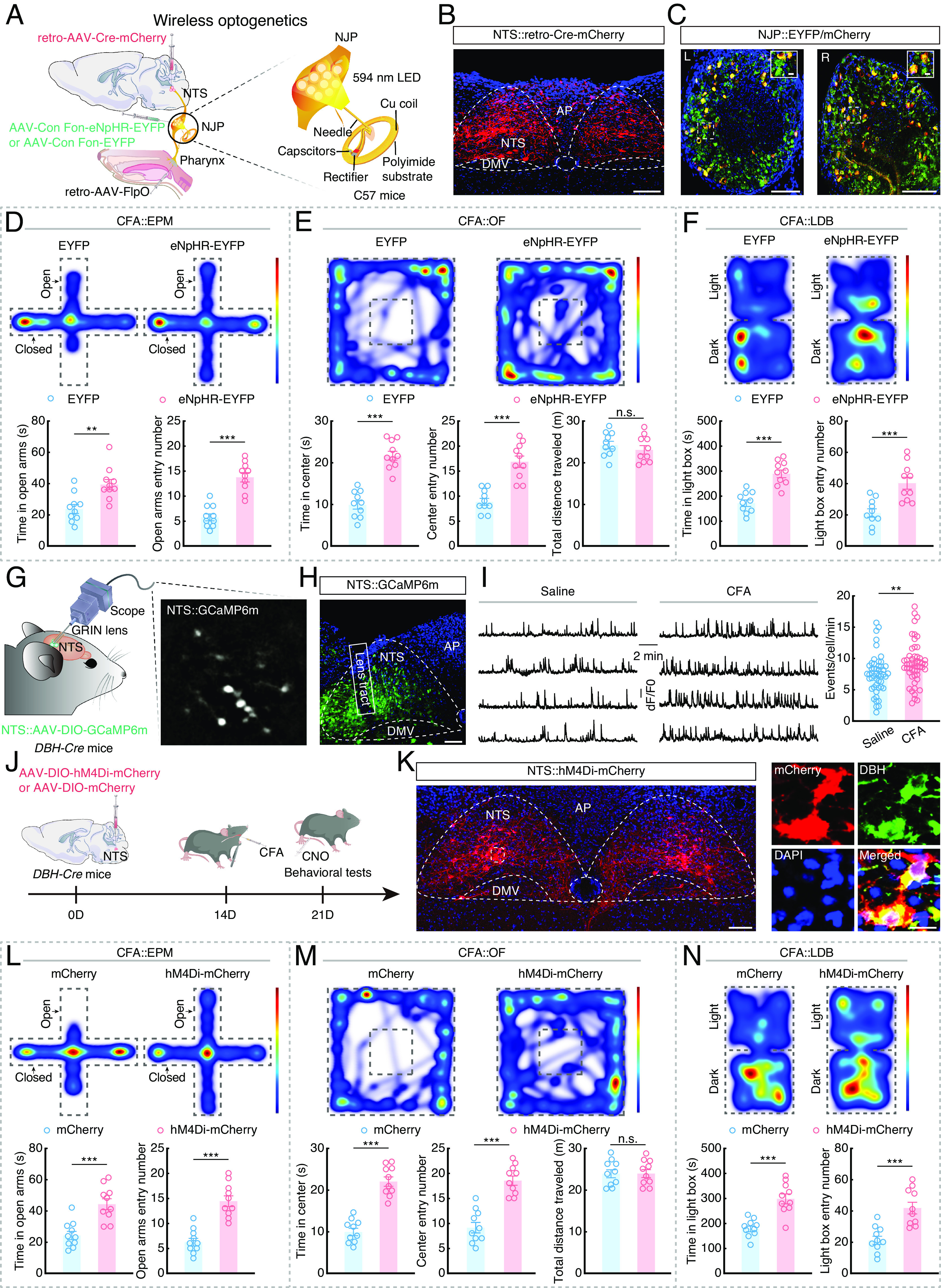
The pharynx→NJP→NTS^NE^ circuit controls pharyngeal inflammation–induced anxiety. (*A*) Schematic of the viral injection and wireless optogenetics. (*B*) Typical image showing the infusion site within the NTS. (Scale bar, 100 μm.) (*C*) Typical images showing the infusion site within NJP superganglia. (Scale bars, 100 μm.) The white boxes depict the areas shown in the boxes of the NJP superganglia. (Scale bars, 10 μm.) L: *Left*; R: *Right*. (*D*) Representative heatmaps of the travel trajectory and summarized data for the EPM test with EYFP and eNpHR-EYFP CFA mice. (*n* = 10 mice per group; *Left*, *t*_18_ = 3.578, *P* = 0.0022; *Right*, *t*_18_ = 6.934, *P* < 0.0001). (*E*) Representative heatmaps of the travel trajectory and summarized data for the OF test with EYFP and eNpHR-EYFP CFA mice. (*n* = 10 mice per group; *Left*, *t*_18_ = 8.093, *P* < 0.0001; *Middle*, *t*_18_ = 6.000, *P* < 0.0001; *Right*, *t*_18_ = 0.8318, *P* = 0.4164). (*F*) Representative heatmaps of the travel trajectory and summarized data for the LDB test with EYFP and eNpHR-EYFP CFA mice. (*n* = 10 mice per group; *Left*, *t*_18_= 5.794, *P* < 0.0001; *Right*, *t*_18_ = 4.090, *P* = 0.0007). (*G*) Schematic of the viral injection and microendoscope imaging in freely moving mice. (*H*) Representative image showing the expression of GCaMP6m and the GRIN lens tract in the NTS. (Scale bar, 100 µm.) (*I*) Sample traces (*Left*) and summarized data (*Right*) showing the spontaneous Ca^2+^ transient frequency of NTS^NE^ neurons from saline and CFA mice. (Saline, *n* = 49 cells; CFA, *n* = 51 cells; *U* = 831, *P* = 0.0036). (*J*) Schematic of viral injection and chemogenetic inhibition design. (*K*) Representative image showing the expression of hM4Di-mCherry in the NTS (*Left*) and predominantly colocalized with DBH antibody (*Right*). (Scale bars, 100 µm [*Left*] or 20 µm [*Right*].) (*L*) Representative heatmaps of the travel trajectory and summarized data for the EPM test with mCherry and hM4Di-mCherry CFA mice (after injection of CNO; *n* = 10 mice per group; *Left*, *t*_18_ = 4.591, *P* = 0.0002; *Right*, *t*_18_ = 6.252, *P* < 0.0001). (*M*) Representative heatmaps of the travel trajectory and summarized data for the OF test with mCherry and hM4Di-mCherry CFA mice (after injection of CNO; *n* = 10 mice per group; *Left*, *t*_18_ = 8.436, *P* < 0.0001; *Middle*, *t*_18_ = 6.757, *P* < 0.0001; *Right*, *t*_18_ = 0.03862, *P* = 0.9696). (*N*) Representative heatmaps of the travel trajectory and summarized data for the LDB test with mCherry and hM4Di-mCherry CFA mice (after injection of CNO; *n* = 10 mice per group; *Left*, *t*_18_ = 5.315, *P* < 0.0001; *Right*, *t*_18_ = 4.551, *P* = 0.0002). Significance was assessed by Mann–Whitney *U* test in (*I*) and two-tailed unpaired Student’s *t* tests in (*D*, *E*, *F*, *L*, *M*, and *N*). All data are presented as the mean ± SEM. **P < 0.01; ***P < 0.001, n.s., not significant.

We next investigated how this pharynx→NJP→NTS circuit functions in pharyngeal inflammation–induced anxiety-like behaviors by injecting retro-hSyn-FlpO into the pharynx, a *Cre* and Flp-dependent AAV carrying eNpHR-enhanced yellow fluorescent protein (AAV-hSyn-Con Fon-eNpHR-EYFP) into the bilateral NJP ganglia, and retro-AAV-hSyn-Cre-mCherry into the bilateral NTS. Flexible, wireless optoelectronic implants were placed on the bilateral NJP superganglia of C57 mice (Fig. 3 *A–C*). After 3 wk of virus expression, upon 594 nm light inhibition of NJP neurons, anxiety-like behaviors were significantly alleviated in CFA mice (i.e., more time in and entries into open arms, center zone and lighted area of the EPM, OF, and LDB, respectively), compared with mice expressing the control virus (Fig. 3 *D–F*). Additionally, we performed optogenetic inhibition of NJP terminals by infusion of the NJP with a *Cre*-dependent AAV carrying eNpHR (AAV-DIO-eNpHR-mCherry) and then inhibited the NTS with yellow light (594 nm) delivered by an optical fiber in *VgluT2-Cre* mice with CFA injected in the pharynx (*SI Appendix*, Fig. S6 *A–C*). Behavioral assays revealed that optogenetic inhibition of eNpHR-expressing NJP terminals in the NTS resulted in significantly alleviating anxiety-like behaviors in mice with CFA-induced pharyngitis (*SI Appendix*, Fig. S6 *D–F*).

Immunofluorescence staining showed that c-Fos levels were significantly elevated in the NTS at 7 d after CFA injection compared to that in controls (*SI Appendix*, Fig. S7 *A*, *B*, and *D*). CFA-induced increased c-Fos in the NTS was largely abolished by transecting the glossopharyngeal and vagal nerves (*SI Appendix*, Fig. S7 *C* and *D*). In addition, in CFA mice without nerve transection, ~62% of c-Fos cells that are DBH positive and ~79% of DBH cells that are c-Fos positive (*SI Appendix*, Fig. S7 *E and F*).

To selectively monitor the response of NTS^NE^ neurons in CFA mice at single-neuron resolution, we infused an AAV expressing the *Cre*-dependent fluorescent Ca^2+^ indicator GCaMP6m (AAV-DIO-GCaMP6m) into the NTS of *DBH-Cre* mice (which specifically express *Cre* recombinase in norepinephrinergic neurons), and mounted a microendoscopic gradient index (GRIN) lens at the top of the NTS (Fig. 3 *G* and *H*). In freely moving mice, the Ca^2+^ transient frequency was significantly higher in NTS^NE^ neurons with CFA injection for 7 d (Fig. 3*I* and Movie S1). These results led us to examine whether chemogenetic inhibition of these neurons could affect anxiety-like behaviors. For this purpose, an AAV expressing *Cre*-dependent inhibitory hM4Di (AAV-DIO-hM4Di-mCherry) was infused into the NTS of *DBH-Cre* mice, and its ligand clozapine-N-oxide (CNO) were intraperitoneally injected to inhibit NTS^NE^ neuronal activity (Fig. 3 *J* and *K*). EPM, OF, and LDB tests showed that inhibiting NTS^NE^ neurons resulted in significantly relieving anxiety-like behaviors of CFA mice (Fig. 3 *L–N*). Taken together, these data indicated that the pharynx→NJP→NTS^NE^ circuit is required for pharyngeal inflammation–induced anxiety.

### Defining Functional Connections of a NTS^NE^→vBNST Circuit.

**Fig. 4.**
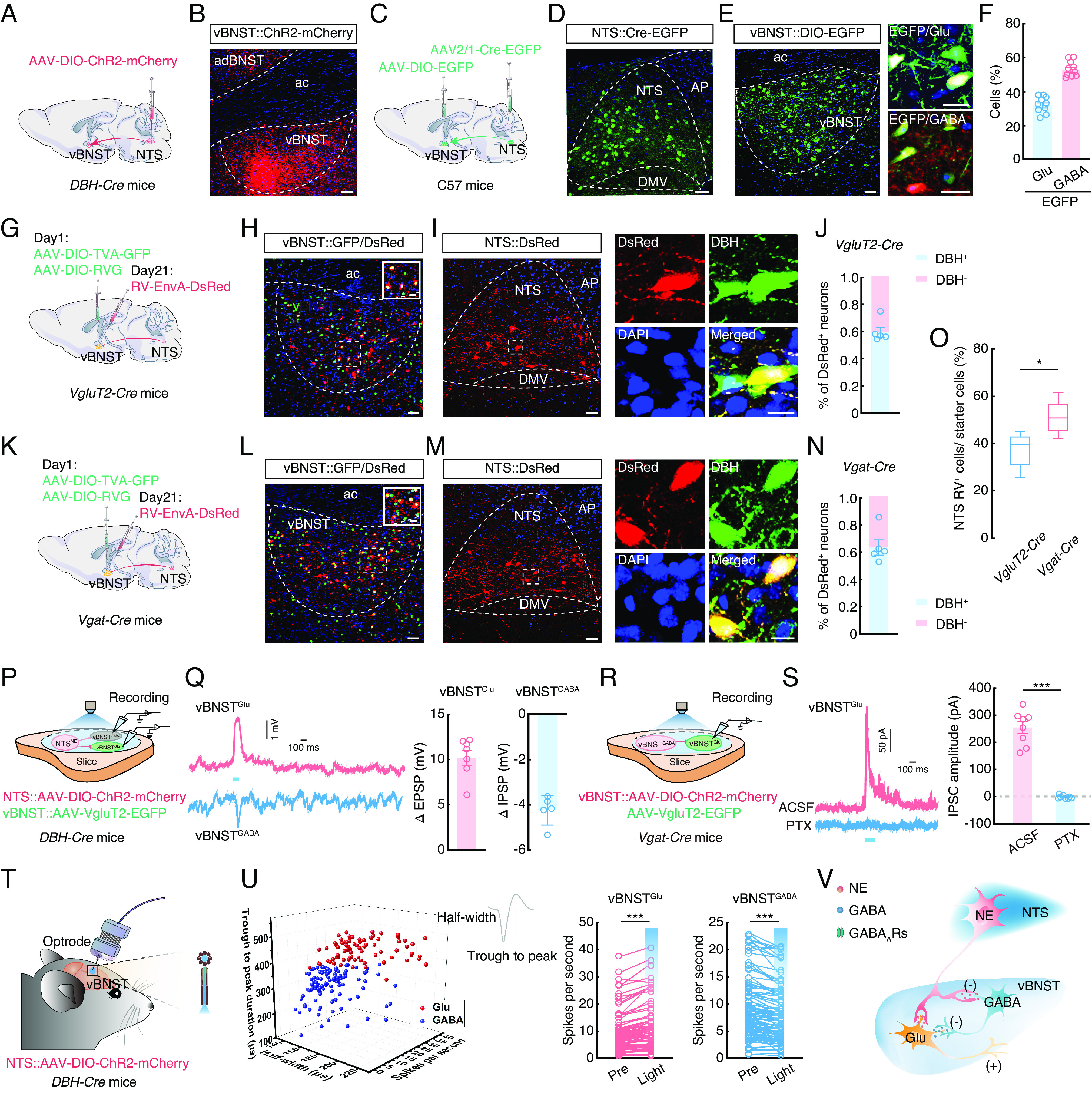
Defining functional connections of the NTS^NE^→vBNST circuit. (*A*) Schematic of the viral injection. (*B*) Representative image of mCherry^+^ fiber expression in the ventral bed nucleus of the stria terminalis (vBNST) of *DBH-Cre* mice. (Scale bar, 50 µm). (*C*) Schematic of the viral injection. (*D*) Representative images showing the infusion site within the NTS. (Scale bar, 50 µm.) (*E*) Representative images showing the infusion site within the vBNST (*Left*) and EGFP-labeled neurons within the vBNST colocalize with glutamate or GABA antibody (*Right*). (Scale bars, 50 µm [*Left*] or 20 µm [*Right*].) (*F*) Statistics showing that EGFP-labeled neurons within the vBNST colocalized with glutamate and GABA antibody (*n* = 10 slices from five mice). (*G*) Schematic of the viral injection. (*H*) Representative images showing the infusion site within the vBNST of *VgluT2-Cre* mice. Starter cells (yellow) coexpress AAV-DIO-TVA-GFP, AAV-DIO-RVG (green), and RV-EnvA-ΔG-DsRed (red). (Scale bar, 50 µm.) The white box depicts the area shown in the box of the vBNST. (Scale bar, 20 µm.) (*I*) Representative images of the DsRed^+^ neurons in the NTS and colocalized with the DBH antibody of *VgluT2-Cre* mice. (Scale bars, 50 µm [*Left*] or 20 µm [*Right*].) (*J*) Quantitative analysis showing ~60% of DsRed^+^ neurons colocalized with DBH antibody signal in the NTS for cell type–specific retrograde trans-monosynaptic tracing of projections to the vBNST in *VgluT2-Cre* mice. (*K*) Schematic of the viral injection. (*L*) Representative images showing the infusion site within the vBNST of *Vgat-Cre* mice. Starter cells (yellow) coexpress AAV-DIO-TVA-GFP, AAV-DIO-RVG (green), and RV-EnvA-ΔG-DsRed (red). (Scale bar, 50 µm.) The white box depicts the area shown in the box of the vBNST. (Scale bar, 20 µm.) (*M*) Representative images showing the DsRed^+^ neurons in the NTS and colocalized with the DBH antibody of *Vgat-Cre* mice. (Scale bars, 50 µm [*Left*] or 20 µm [*Right*].) (*N*) Quantitative analysis showing ~64% of DsRed^+^ neurons colocalized with DBH antibody in the NTS for cell type–specific retrograde trans-monosynaptic tracing of projections to the vBNST in *Vgat-Cre* mice. (*O*) Statistical data showing the ratio of RV^+^ neurons in the NTS to starter cells in the vBNST of *VgluT2-Cre* and *Vgat-Cre* mice (*n* = 5 images from five mice per group; *t_8_ =* 2.936*, P* = 0.0188). (*P*) Schematic of the viral injection and recording configuration in acute slices. (*Q*) Representative traces (*Left*) and summarized data (*Right*) recorded from neurons after photostimulation (473 nm light, 100 ms, blue bar) of NTS^NE^ fibers (ΔEPSP, *n* = 7 cells, ΔIPSP, *n* = 5 cells). (*R*) Schematic of the viral injection and recording configuration in acute slices. (*S*) Representative traces (*Left*) and summarized data (*Right*) recorded from the vBNST^Glu^ of light-evoked currents (473 nm light, 100 ms, blue bar) before and after PTX (picrotoxin, 10 µM) treatment (*n* = 8 cells, *t*_7_ = 10.97, *P* < 0.0001). (*T*) Schematic of the viral injection and multitetrode recording in freely moving mice. (*U*) All recorded light-sensitive neurons (*n* = 189) were classified as wide-spiking putative glutamatergic cells (red, *n* = 81) and GABAergic cells (blue, *n* = 108) (*Left*). Summarized data (*Right*) recorded from the neurons in vBNST before and during light photostimulation of NTS^NE^ fibers (vBNST^Glu^, *t*_80_ = 8.895, *P* < 0.0001; vBNST^GABA^, *t*_107_ = 9.522, *P* < 0.0001). (*V*) Schematic of NTS^NE^ projections onto vBNST. EPSP, excitatory postsynaptic potential. IPSP, inhibitory postsynaptic potential. IPSC, inhibitory postsynaptic current. Significance was assessed by two-tailed unpaired Student’s *t* tests in (*O)* and two-tailed paired Student’s *t* tests in (*S* and *U*). All data are presented as the mean ± SEM. *P < 0.05, ***P < 0.001.

We next sought to identify the downstream regions of the NTS^NE^ potentially involved in mediating anxiety-like behaviors. Briefly, 3 wk after the NTS infusion of AAV-DIO-ChR2-mCherry in *DBH-Cre* mice (Fig. 4*A* and *SI Appendix*, Fig. S8 *A* and *B*), we detected mCherry^+^ fibers in multiple brain regions previously shown to participate in anxiety, including the ventral bed nucleus of the stria terminalis (vBNST), parabrachial nucleus (PB), central nucleus of the amygdala (CeA), ventrolateral periaqueductal gray (vlPAG), locus coeruleus (LC), paraventricular hypothalamic nucleus (PVN), and paraventricular thalamic nucleus (PVT) (Fig. 4*B* and *SI Appendix*, Fig. S8 *C–I*). Among these nuclei, the vBNST had the highest density of mCherry^+^ fibers (*SI Appendix*, Fig. S8*J*).

NTS^NE^ neurons project to several other areas that have been previously associated with anxiety (27, 28). Manipulating the NTS→vBNST circuit could possibly affect activity in other regions targeted by collateral projections from the same NTS neuron. We therefore injected a retro-AAV-Cre virus into the vBNST and an AAV-DIO-ChR2-mCherry into the NTS of C57 mice to identify collateral projections of vBNST-projecting NTS neurons (*SI Appendix*, Fig. S9 *A* and *B*). We found that vBNST-projecting NTS neurons also innervate the PB, CeA, vlPAG, LC, PVN, and PVT, but with relatively few fibers (*SI Appendix*, Fig. S9 *A* and *C*).

To characterize the NJP→NTS→vBNST circuit, we used a triple tracing approach in mice by injecting retro-AAV-mCherry into the vBNST, AAV2/1-Cre into the NJP, and AAV-DIO-EGFP into the NTS (*SI Appendix*, Fig. S10 *A* and *B*). We identified mCherry^+^ EGFP^+^ neurons in the NTS and found that ~65% of the NTS neurons that received inputs from the NJP also projected to the vBNST (*SI Appendix*, Fig. S10 *C* and *D*).

To identify the NTS targets within the vBNST, we injected an AAV2/1-Cre-EGFP virus into the NTS and an AAV-DIO-EGFP virus into the vBNST of C57 mice (Fig. 4 *C–E*) and 3 wk later detected EGFP-expressing neurons in the vBNST that colocalized with ~32% of Glu^+^ neurons and ~53% of GABA^+^ neurons (Fig. 4 *E* and *F*). We then conducted cell type–specific retrograde trans-monosynaptic tracing by infusing the vBNST of *VgluT2-Cre* mice, which selectively express *Cre* recombinase in glutamatergic neurons, with *Cre*-dependent helper viruses and rabies virus (RV) (Fig. 4 *G* and *H* and *SI Appendix*, Fig. S11 *A–C*). We observed DsRed-positive neurons in several regions, including the NTS (*SI Appendix,* Fig. S11 *D–F*), that ~60% colocalized with signals from DBH antibody (Fig. 4 *I* and *J*). A similar tracing strategy in *Vgat-Cre* mice, which selectively express *Cre* recombinase in GABAergic neurons, showed that ~64% of GABAergic neurons in vBNST (vBNST^GABA^) also received inputs from the NTS and colocalized with DBH antibody signal (Fig. 4 *K–N* and *SI Appendix*, Fig. S12 *A* and *B*). We also observed DsRed-positive neurons in several other regions (*SI Appendix,* Fig. S12*C*). Additionally, the ratio of RV^+^ NTS cells to vBNST starter cells was ~38% in *VgluT2-Cre* mice and ~52% in *Vgat-Cre* mice (Fig. 4*O*), collectively suggesting that NTS^NE^ preferentially project to vBNST^GABA^ neurons.

To better understand the activity of these NTS^NE^→vBNST connections, we infused the NTS of *DBH-Cre* mice with AAV-DIO-ChR2-mCherry and infused the vBNST with AAV-VgluT2-EGFP (Fig. 4*P*). Based on the preponderance of direct projections from NTS^NE^→vBNST^GABA^, we detected postsynaptic potentials in the vBNST^GABA^ neurons and vBNST^Glu^ neurons following blue light stimulation (473 nm) of ChR2-expressing NTS^NE^ axon terminals in the vBNST. We found that innervation by these NTS^NE^ resulted in increased inhibitory postsynaptic potentials (IPSPs) in vBNST^GABA^ neurons (Fig. 4*Q* and *SI Appendix*, Fig. S13 *A* and *B*), but increased excitatory postsynaptic potentials (EPSPs) in vBNST^Glu^ neurons. These results suggested the possible presence of a microcircuit (NTS^NE^ projections inhibit vBNST^GABA^, and subsequently disinhibit vBNST^Glu^) among different neuron types within the vBNST. To test this possibility, we then injected vBNST of *Vgat-Cre* mice with AAV-DIO-ChR2-mCherry and noted that blue light stimulation of local vBNST^GABA^ neurons led to increased inhibitory postsynaptic currents (IPSCs) in vBNST^Glu^, which could be abolished by treatment with the GABA receptor antagonist, picrotoxin (Fig. 4 *R* and *S* and *SI Appendix*, Fig. S13 *C* and *D*). These results supported the role of a vBNST^GABA→Glu^ microcircuit in responsive to innervation by NTS^NE^ neurons.

Subsequent in vivo multitetrode recordings further showed that optical activation of NTS^NE^ terminals in the vBNST resulted in an increased firing rate of vBNST^Glu^ neurons and a decreased firing rate of vBNST^GABA^ neurons (Fig. 4 *T* and *U*) The above experimental results supported a microcircuit organization in which vBNST^Glu^ neurons are innervated by local vBNST^GABA^ interneurons. These results indicated the presence of multiple functional connections within the NTS→vBNST circuit, including 1) NTS^NE^ inputs that inhibit vBNST^GABA^ interneurons, which in turn project to and inhibit vBNST^Glu^ neurons, forming a local microcircuit, and 2) direct NTS^NE^ projections to vBNST^Glu^ neurons (Fig. 4*V*).

### Inhibition of the NJP→NTS^NE^→vBNST Circuit Reduces Pharyngeal Inflammation–Induced Anxiety.

**Fig. 5.**
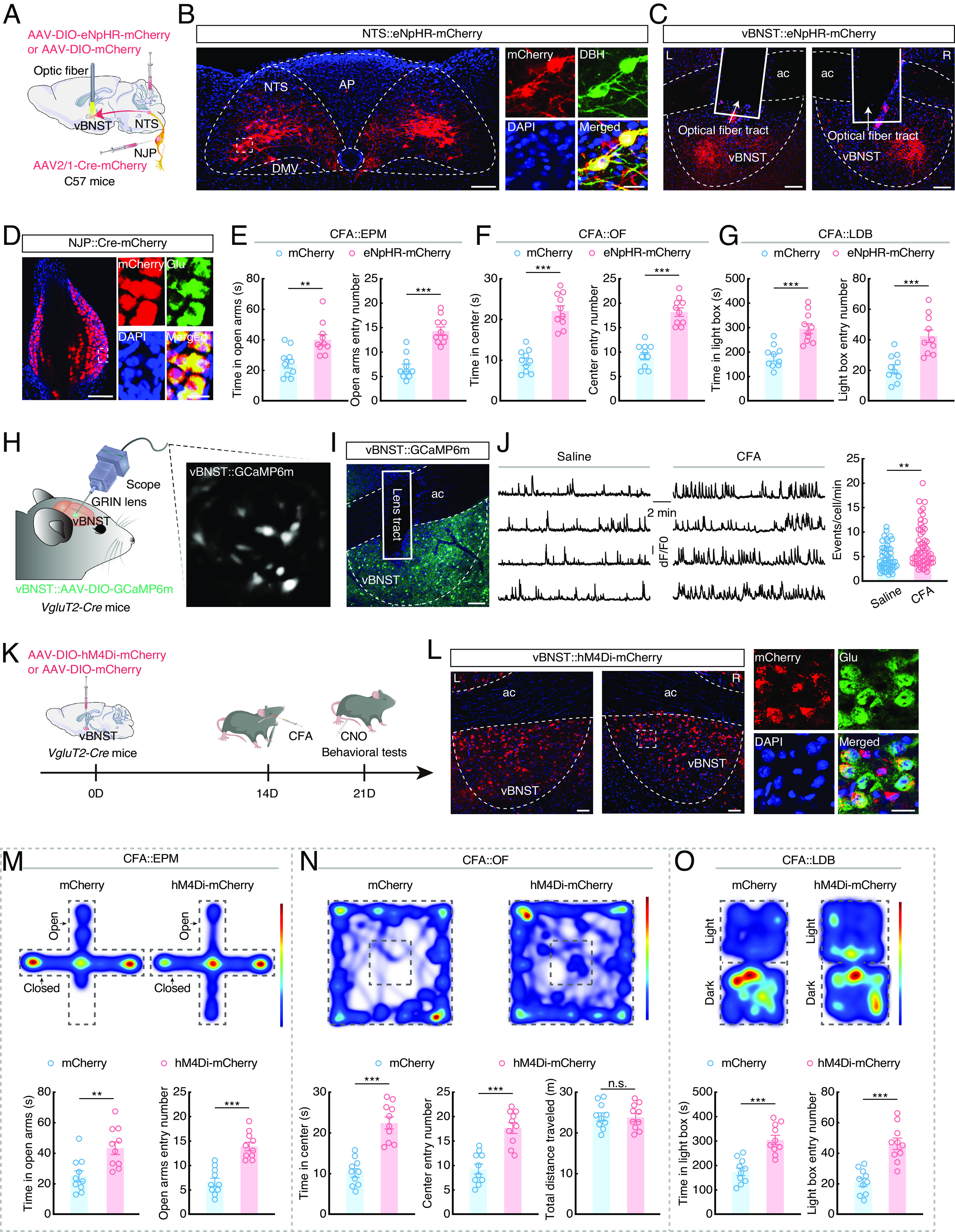
Inhibiting the NJP→NTS^NE^→vBNST circuit can reduce anxiety-like behaviors in CFA mice. (*A*) Schematic of the optogenetic inhibition of the NJP→NTS^NE^→vBNST circuit. (*B*) Representative images showing the expression of eNpHR-mCherry in the NTS, and mCherry^+^ neurons being detected by DBH antibody. (Scale bars, 100 µm [*Left*] or 20 µm [*Right*].) (*C*) Representative images of the projection fibers and optical fiber placement in the vBNST. (Scale bars, 100 µm.) L: *Left*; R: right. (*D*) Representative images showing the expression of virus in NJP neurons (*Left*) and mCherry^+^ neurons colocalized with glutamate antibody (*Right*). (Scale bars, 50 µm [*Left*] or 20 µm [*Right*].) (*E*) Summarized data for the EPM test with mCherry and eNpHR-mCherry CFA mice (*n* = 10 mice per group; *Left*, *t*_18_ = 3.518, *P* = 0.0025; *Right*, *t*_18_ = 6.460, *P* < 0.0001). (*F*) Summarized data for the OF test with mCherry and eNpHR-mCherry CFA mice (*n* = 0 mice per group; *Left*, *t*_18_ = 7.908, *P* < 0.0001; *Right*, *t*_18_ = 7.853, *P* < 0.0001). (*G*) Summarized data for the LDB test with mCherry and eNpHR-mCherry CFA mice (*n* = 10 mice per group; *Left*, *t*_18_ = 4.868, *P* = 0.0001; *Right*, *t*_18_ = 4.292, *P* = 0.0004). (*H*) Schematic of the viral injection and microendoscopic imaging in freely moving mice. (*I*) Representative image showing the expression of GCaMP6m and the GRIN lens tract in the vBNST. (Scale bar, 100 µm.) (*J*) Sample traces (*Left*) and summarized data (*Right*) showing the spontaneous Ca^2+^ transient frequency of vBNST^Glu^ neurons from saline and CFA mice (Saline, *n* = 50 cells; CFA, *n* = 57 cells; *U* = 913, *P* = 0.0012). (*K*) Schematic of viral injection and chemogenetic inhibition design. (*L*) Representative images showing the expression of hM4Di-mCherry in the vBNST (*Left*) and colocalized with glutamate antibody (*Right*). (Scale bars, 50 µm [*Left*] or 20 µm [*Right*].) L: *Left*; R: *Right*. (*M*) Representative heatmaps of the travel trajectory and summarized data for the EPM test with mCherry and hM4Di-mCherry CFA mice (after injection of CNO; *n* = 10 mice per group; *Left*, *t*_18_ = 3.360, *P* = 0.0035; *Right*, *t*_18_ = 6.072, *P* < 0.0001). (*N*) Representative heatmaps of the travel trajectory and summarized data for the OF test with mCherry and hM4Di-mCherry CFA mice (after injection of CNO; *n* = 10 mice per group; *Left*, *t*_18_ = 6.771, *P* < 0.0001; *Middle*, *t*_18_ = 5.607, *P* < 0.0001; *Right*, *t*_18_ = 0.1231, *P* = 0.9034). (*O*) Representative heatmaps of the travel trajectory and summarized data for the LDB test with mCherry and hM4Di-mCherry CFA mice (after injection of CNO; *n* = 10 mice per group; *Left*, *t*_18_= 5.282, *P* < 0.0001; *Right*, *t*_18_ = 5.426, *P* < 0.0001). Significance was assessed by two-tailed unpaired Student’s *t* tests in (*E–G* and *M–O*), and Mann–Whitney *U* test in (*J*). All data are presented as the mean ± SEM. **P < 0.01; ***P < 0.001, n.s., not significant.

We then investigated the potential role of this NJP→NTS^NE^→vBNST circuit in anxiety-like behaviors in CFA mice. To this end, we conducted optical inhibition of NTS^NE^→vBNST by infusing the NTS with AAV-DIO-eNpHR-mCherry and implanting optical fibers in the bilateral vBNST of *DBH-Cre* mice with CFA-induced pharyngitis (*SI Appendix*, Fig. S14 *A–C*). Optical inhibition resulted in significant alleviation of anxiety-like behaviors in CFA mice (*SI Appendix*, Fig. S14 *D–F*). We then modulated the activity of the NJP→NTS→vBNST triple circuit by infusing the bilateral NJP superganglia with AAV2/1-hSyn-Cre-mCherry and injecting the bilateral NTS with AAV-DIO-eNpHR-mCherry and implanting optical fibers in the bilateral vBNST of C57 mice (Fig. 5 *A–D*). Optical inhibition resulted in significant alleviation of anxiety-like behaviors in CFA mice (Fig. 5 *E–G* and *SI Appendix*, Fig. S15), these findings supporting the involvement of the NJP→NTS^NE^→vBNST circuit in alleviating pharyngeal inflammation–induced anxiety.

To examine the neuronal activity of glutamate and GABA neurons in the vBNST, we selectively monitored the response of these neurons to pharyngeal inflammation at single-neuron resolution. To this end, AAV-DIO-GCaMP6m was infused into the vBNST of *VgluT2-Cre* or *Vgat-Cre* mice, and a GRIN lens was positioned above the vBNST (Fig. 5 *H* and *I* and *SI Appendix*, Fig. S16 *A* and *B*). Following CFA injection of the pharynx, Ca^2+^ transient frequency significantly increased in vBNST^Glu^ neurons in freely moving mice, but significantly decreased in vBNST^GABA^ neurons, compared to that in saline control mice (Fig. 5*J* and *SI Appendix*, Fig. S16*C* and Movie S2).

Chemogenetic inhibition of vBNST^Glu^ neurons resulted in significantly alleviating anxiety-like behaviors in *VgluT2-Cre* CFA mice compared with mice expressing the control virus (Fig. 5 *K–O*). In addition, we injected AAV-expressing *Cre*-dependent excitatory hM3Dq (AAV-DIO-hM3Dq-mCherry) in the vBNST with intraperitoneal injection of CNO to activate vBNST^GABA^ neurons in *Vgat-Cre* mice (*SI Appendix*, Fig. S16 *D* and *E*). In these CFA mice, activation of vBNST^GABA^ neurons again resulted in a significant alleviation of anxiety-like behaviors (*SI Appendix*, Fig. S16 *F–H*).

vBNST has been found to contain multiple cell type populations beyond excitatory/inhibitory populations, such as enkephalin (ENK) and corticotropin-releasing factor (CRF), nociceptin (NOC), and neuropeptide Y (NPY). which are important for anxiety-like behaviors (29–33). To evaluate the NTS-innervated vBNST subpopulation neurons that potentially play a role in pharyngeal inflammation–induced anxiety behaviors, we conducted immunofluorescence staining after injected AAV2/1-Cre-EGFP into the NTS and AAV-DIO-EGFP into the vBNST in C57 mice (*SI Appendix*, Fig. S17 *A–C*). At 21 d after injection, ~40% of the EGFP^+^ cells in the vBNST were positive for neuropeptide precursor, proenkephalin (PENK, which encodes a preproprotein for ENK) (*SI Appendix*, Fig. S17*D*), ~38% were positive for CRF (*SI Appendix*, Fig. S17*E*), ~9% were positive for NPY (*SI Appendix*, Fig. S17*F*), and ~6% were positive for NOC (*SI Appendix*, Fig. S17*G*). Additionally, ~78% of the NTS→vBNST^ENK^ cells were positive for GABA (*SI Appendix*, Fig. S18 *A* and *B*), and chemogenetic activation of the vBNST^ENK^ neurons resulted in significant alleviation of anxiety-like behaviors (*SI Appendix*, Fig. S18 *C–G*). ~60% of the NTS→vBNST^CRF^ cells were positive for glutamate (*SI Appendix*, Fig. S19 *A* and *B*), and chemogenetic inhibition of the vBNST^CRF^ neurons resulted in significant alleviation of anxiety-like behaviors (*SI Appendix*, Fig. S19 *C–G*). Thus, these findings demonstrate that NTS-innervated vBNST^ENK^ and vBNST^CRF^ neurons contribute directly to anxiety induced by pharyngeal inflammation.

### Pharyngeal Inflammation Induces Anxiety-Like Behaviors Through a Pharynx-to-Brain Axis.

**Fig. 6.**
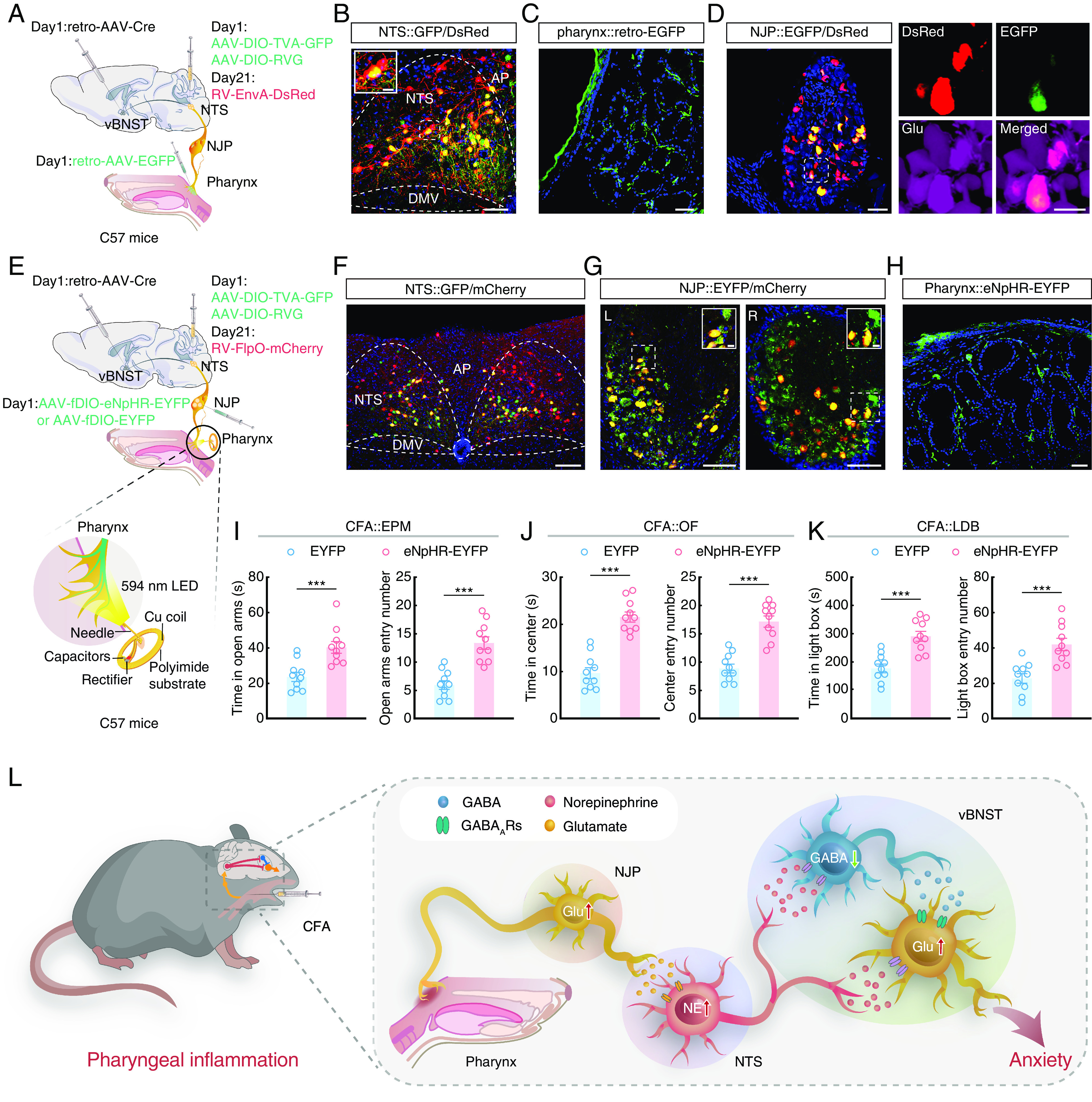
Pharyngeal inflammation induces anxiety-like behaviors through a pharynx-to-brain axis in mice. (*A*) Schematic of the quadruple tracing strategy. (*B*) Representative images of the starter neurons (yellow) within the NTS. (Scale bar, 50 μm.) The white box depicts the area shown in the box of the NTS. (Scale bar, 10 μm.) (*C*) Representative image of the infusion site within the pharynx. (Scale bar, 50 μm.) (*D*) Representative confocal images showing DsRed^+^ from NTS colabeled with EGFP^+^ neurons innervating the pharynx (*Left*). The confocal neurons were colocalized with a glutamate antibody (*Right*). (Scale bars, 50 μm [*Left*] or 20 μm [*Right*].) (*E*) Schematic of the viral injection and wireless optogenetics. (*F*) Representative image showing the expression of virus in the NTS. (Scale bar, 100 μm.) (*G*) Representative images showing the EYFP^+^ neurons colocalized with mCherry^+^ in NJP superganglia. (Scale bars, 100 μm.) The white boxes depict the areas shown in the boxes of the NJP superganglia. (Scale bars, 20 μm.) L: *Left*; R: *Right*. (*H*) Representative image showing the projection fibers in the pharynx. (Scale bar, 50 μm.) (*I*) Summarized data for the EPM test with EYFP and eNpHR-EYFP CFA mice (594 nm light, *n* = 10 mice per group; *Left*, *t*_18_ = 3.946, *P* = 0.0009; *Right*, *t*_18_ = 5.526, *P* < 0.0001). (*J*) Summarized data for the OF test with EYFP and eNpHR-EYFP CFA mice (594 nm light, *n* = 10 mice per group; *Left*, *t*_18_ = 7.516, *P* < 0.0001; *Right*, *t*_18_ = 6.605, *P* < 0.0001). (*K*) Summarized data for the LDB test with EYFP and eNpHR-EYFP CFA mice (594 nm light, *n* = 10 mice per group; *Left*, *t*_18_ = 4.868, *P* = 0.0001; *Right*, *t*_18_ = 4.347, *P* = 0.0004). (*L*) Schematic showing a pharynx-to-brain axis mediating pharyngeal inflammation–induced anxiety. Significance was assessed by two-tailed unpaired Student’s *t* tests in (*I–K*). All data are presented as the mean ± SEM. ***P < 0.001.

We then investigated the structure of connections in the pharynx→NJP→NTS^NE^→vBNST circuit. For this purpose, we infused retro-AAV-Cre into the vBNST, *Cr*e-dependent helper viruses and RV into the NTS, and retro-hSyn-EYFP into the pharynx (Fig. 6 *A–C*). We then observed that EGFP^+^ DsRed^+^ colabeled neurons in the NJP also colocalized with glutamate antibody (Fig. 6*D*), thus supporting the presence of a pharynx→NJP→NTS^NE^→vBNST circuit.

Wireless optogenetic neuronal inhibition was employed to better understand the function of this pharynx→NJP→NTS^NE^→vBNST circuit. For these behavioral experiments, retro-AAV-Cre was infused into the bilateral vBNST, *Cre*-dependent helper viruses and a retrograde trans-monosynaptic virus RV-FlpO was injected into the bilateral NTS, and AAV-fDIO-eNpHR-EYFP was injected into the bilateral NJP superganglia of mice so that only the NJP neurons projecting to the NTS→vBNST circuit could express the AAV-fDIO-eNpHR-EYFP viruses. Flexible wireless optic fibers were implanted at the pharynx (Fig. 6 *E–H*). After 3 wk, we found that CFA mice with optical inhibition of the pharynx→NJP→NTS^NE^→vBNST circuit exhibited alleviated anxiety-like behaviors (Fig. 6 *I–K* and *SI Appendix*, Fig. S20) compared with NJP superganglia infusion of control virus AAV-fDIO-EYFP. These data suggested that inhibiting the pharynx-to-brain axis could alleviate pharyngeal inflammation–induced anxiety-like behaviors in mice (Fig. 6*L* and *SI Appendix,* Fig. S21).

## Discussion

Although the phenomenon of anxiety among patients with pharyngitis has been well described in previous clinic studies (34, 35), the precise cell type–specific organization and functional pathways responsible for modulating anxiety through this pharynx-to-brain axis have remained unknown. This study defines a pharynx-to-brain axis (pharynx→NJP→NTS^NE^→vBNST) through which pharyngeal inflammation can induce anxiety-like behaviors in mice. Central to these processes are circuit mechanisms that require activation of inputs from the pharynx to NJP neurons, which in turn project to and activate NTS^NE^ neurons, and innervate vBNST neurons in CFA mice. This pharynx-to-brain axis mechanistically links pharyngeal inflammation and emotional response and suggests potential therapeutic targets for pharyngitis-associated anxiety.

The pharynx serves as the entrance to the respiratory and digestive tracts (36). Once the pharynx is irritated (e.g., inflammation and injury), its barrier function may be disrupted, ultimately resulting in complex physiological disorders (37). While sensory transmission from peripheral organs through the NJP superganglia has received research attention (38), the role of the NJP superganglia in anxiety has been overlooked. Our study depicts a pharynx→NJP→NTS^NE^ circuit through cell type–specific viral tracing strategies. Findings in our current study firmly link the afferent function of the NJP neurons with mood disorder under inflammatory conditions in the pharynx.

The NTS has been identified as a hub for integrating visceral sensory signals to the brain (39–43). The brain regions respond to peripheral sensory or inflammatory signals transmitted from the NTS to trigger anxiety-associated behaviors remains elusive. Our viral tracing experiments identified functional connections that form an NTS→vBNST circuit, and specifically inhibiting NTS^NE^ inputs to the vBNST could alleviate anxiety-like behaviors in CFA mice. These findings support that NTS^NE^ neurons may serve as a bridge between inflammatory signaling in visceral organs and the vBNST to promote an emotional response.

Anterograde tracing identified several regions that receive fewer projections than the vBNST from the NTS, while retrograde tracing identified regions other than the NTS that also project to vBNST. Among these various regions, the CeA was detected in both groups. Given that CeA consists of 95% GABA neurons (44–46), it is therefore possible that the NTS could innervate CeA^GABA^ neurons, which in turn suppress vBNST^GABA^, which could ultimately contribute to the observed differences in postsynaptic currents between vBNST^GABA^ and vBNST^Glu^ neurons.

Our viral tracing results showed that the NTS^NE^ directly projects to vBNST^Glu^, although inhibition of NTS^NE^ inputs to the vBNST could alleviate anxiety-like behaviors in CFA mice, in fact, we have no data showing that inhibiting NTS^NE^→vBNST while simultaneously blocking the vBNST^GABA→Glu^ microcircuit results in abolishing the observed alleviation of anxiety behaviors. Therefore, we cannot exclude the possibility that modulation by an NTS^NE^→vBNST^Glu^ circuit also affects anxiety behaviors in this pharyngeal inflammation model. However, based on our evidence showing that vBNST^GABA^ neurons appear indispensable to the pharyngitis-induced anxiety-like behaviors, combined with our data showing significantly stronger direct NTS^NE^ innervation of GABAergic, rather than glutamatergic neurons in the vBNST, as well as prominent, direct vBNST^GABA→Glu^ projections, we hypothesize that the greater inhibition of vBNST^GABA^ activity results in blocking the inhibitory microcircuit and disinhibition of vBNST^Glu^ in response to pharyngeal inflammation.

Although we observed a decrease in food intake in the first 3 d after CFA injection, the CFA and control groups showed no significant difference in food intake at 7 d after CFA injection in our study. Previous studies have reported that activation of NTS^NE^ neurons results in anorexia in the first day (47), while protein kinase C-delta (PKC-δ) neurons in the oval region of the BNST are activated by LPS or IL1β and produce anorexia when activated for 2 h at a time, but not after several days of activation (48). Given that both NTS^NE^ and ovBNST^PKC-δ^ neurons appear to be involved in regulating food intake, it is plausible that both of these neuron types may be involved in anorexia in the acute phase (i.e., the first 3 d).

This mechanistic investigation defines a pharynx→​NJP→​NTS^NE^→vBNST^GABA→Glu^ circuit that is implicated in pharyngitis-associated anxiety, highlighting a pharynx-to-brain axis through which inflammation in the pharynx is processed into an anxiety response.

## Materials and Methods

See *SI Appendix*, *Materials and Methods* for additional detailed information about the procedures used in this study. All data necessary to understand and assess the conclusions of this study are available in the main text or *SI Appendix*. There are no restrictions on data availability in the manuscript.

### Human Participants.

This cross-sectional study was approved by the Ethics Committee of the First Affiliated Hospital, University of Science and Technology of China, and all the subjects signed informed consent to participate in the study.

### Animals.

Mice at 8 to 10 wk of age were used. All animal protocols were approved by the Animal Care and Use Committee of the University of Science and Technology of China.

### Animal Model of Pharyngeal Inflammation.

Complete Freund’s Adjuvant (CFA, 5 µL or 10 µL, Sigma) injection into the mucosa of the pharynx with an endoscope was used to conduct a pharyngeal inflammation mouse model.

### Stereotaxic Surgeries and Viral Injections.

Virus was injected into the target area through the glass microelectrode connected to an infusion pump (micro 4, WPI). All mice were transcardially perfused with ice-cold 0.9% saline followed by 4% paraformaldehyde. Images of signal expression were obtained using a confocal microscope (LSM 980, ZEISS, Germany).

### Microendoscopic Imaging and Data Processing.

Integrated microendoscopic GRIN lens (0.5 mm in diameter × 6 mm in length, Inscopix, #1050-002211) were implanted into the target areas of the mice for monitoring of calcium signals. For data analysis, fluorescence videos were processed offline with Inscopix data processing software (version 1.1.6).

### In Vivo Multitetrode Recordings.

Eight movable custom-built tetrode arrays were implanted into the target areas of the mice for extracellular recordings. Neuronal signals were amplified and stored with Neurostudio software (Neurostudio), before exporting to Offline Sorter (Plexon) and Neuroexplorer 4 (Nex Technologies) for offline analysis.

### Statistical Analysis.

The Student’s *t* test, Mann–Whitney *U* test, Pearson’s correlation test or one-way and two-way ANOVA, and post hoc analyses were used. All data are expressed as the mean ± SEM.

## Supplementary Material

Appendix 01 (PDF)

Dataset S01 (XLSX)

Movie S1. *In vivo* microendoscopic calcium imaging of NTS^NE^ neurons in saline (left) and CFA (right) *DBH-Cre* mice, which NTS infusion of AAV-DIO-GCaMP6m.

Movie S2. *In vivo* microendoscopic calcium imaging of vBNST^Glu^ neurons in saline (left) and CFA (right) *VgluT2-Cre* mice, which vBNST infusion of AAV-DIO-GCaMP6m.

## Data Availability

All study data are included in the article and/or supporting information.
