# Supplementary material for: A pharynx-to-brain axis controls pharyngeal inflammation–induced anxiety
Source: Proc Natl Acad Sci U S A. 2024 Mar 6;121(11):e2312136121. doi: 10.1073/pnas.2312136121 (PMC10945766; doi:10.1073/pnas.2312136121)
Supplement: Supplementary file 1 — Appendix 01 (PDF) [file pnas.2312136121.sapp.pdf]

PNAS

Supporting Information for

A pharynx-to-brain axis controls pharyngeal inflammation-induced anxiety.

Wan Zhao[1†], Ke Zhang[1†], Wan-Ying Dong[2†], Hao-Di Tang[2, 3†], Jia-Qiang Sun[1], Ji-Ye Huang[2], Guang-Lun Wan[1], Rui-Rui Guan[1], Xiao-Tao Guo[1], Ping-Kai Cheng[2], Ran Tao[4], Jing-Wu Sun[1]*, Zhi Zhang[2, 5]*, and Xia Zhu[2]*

* Jing-Wu Sun, Zhi Zhang, and Xia Zhu
Email: entsun@ustc.edu.cn; zhizhang@ustc.edu.cn; xiazhu@ustc.edu.cn

This PDF file includes:

Supporting text
Figures S1 to S21
Table S1
Legends for Movies S1 to S2
SI References

Other supporting materials for this manuscript include the following:

Movies S1 to S2

Materials and Method

Human participants.

This cross-sectional study was approved by the Ethics Committee of the First Affiliated Hospital, University of Science and Technology of China, and all the subjects signed informed consent to participate in the study. A total of 76 patients (40 males, 36 females; mean 40.30 ± 10.96 years old), from the otolaryngology outpatient clinic, with pharyngitis, who met the inclusion criteria, were enrolled in this study. The inclusion criteria were as follows: (1) people aged 18 to 60 years; (2) pharyngeal discomfort (*e.g*., sore throat, sensation of a foreign body, dry, throat clearing); and (3) physical examination positive for pharyngitis (*e.g.*, pharyngeal hyperemia, mucopurulent or catarrhal secretions, lymphoid follicles on posterior wall, mucosal atrophy appearing dry, smooth, shiny). Patients with the following conditions were excluded: (1) patients who were unable to understand the scale or were uncooperative; (2) patients presenting with psychiatric symptoms due to other somatic disorders; (3) those with a history of psychiatric illness; (4) those with a history of head and neck surgery or anatomical abnormalities; (5) those with a history of severe systemic disease or cancer; and (6) those with any other clinical condition that is judged by the investigator to be inappropriate for the study. 86 healthy controls (44 males, 42 females; mean 39.74 ± 9.63 years old) without pharyngitis were also included in this study. Inclusion criteria were as follows: (1) people between 18 and 60 years of age; (2) those without any pharyngeal discomfort (*e.g.*, sore throat, foreign body sensation, dry, throat clearing); (3) those without any physical examination positive for pharyngitis (*e.g.*, pharyngeal hyperemia, mucopurulent or catarrhal secretions, lymphoid follicles on the posterior wall, mucosal atrophy appearing dry, smooth, shiny).The exclusion criteria of healthy controls are the same as for the patients with pharyngitis included in the study. We have eliminated related conditioning factors between the two groups. All participants completed the study, and there were no losses.

Animals.

We used C57BL/6J, *VglutT2-Cre*, *Vgat-Cre*, *CRF-Cre* (purchased from Charles River or Jackson Laboratories) and *DBH-Cre* mice (provided by Guoqiang Bi) aged 8-10 weeks. All animals were bred in a specific pathogen-free environment with controlled ambient temperature (22 ± 2°C) and humidity (50% ± 10%) under a 12h light-dark cycle (lights on from 08:00 to 20:00). The mice were group-housed with five per cage in a colony with food and water available ad libitum unless a GRIN lens or tetrode array was implanted. All animal protocols were approved by the Animal Care and Use Committee of the University of Science and Technology of China.

Physical examination of the human pharynx.

Physical examination was performed by observation of the oropharynx and hypopharynx with a stroboscopic laryngoscopy (Xion, Berlin, Germany). According to objective pharyngeal features, a Tonsillo-Pharyngitis Assessment (TPA) was carried out to assess the severity of pharyngitis, with higher scores indicating more severe inflammation. The TPA was conducted using a scale from 0 to 3 for each of the seven signs of pharyngitis: oral temperature, oropharyngeal color, number of oropharyngeal enanthems, number of anterior cervical lymph nodes, maximum size and tenderness of anterior cervical lymph nodes, and tonsillar size. The TPA score was defined as the sum of these seven ratings(1, 2).

Evaluation of anxiety in humans.

The Self-Rating Anxiety Scale (SAS) was used to assess anxiety in the enrolled subjects(3). The SAS consists of 20 questions, each scored as 1-4 points, resulting in a raw score of 20-80 points; the standard score is calculated by integer (1.25 × raw score), where a standard score of >50 is defined as anxiety(4).

Murine model of pharyngeal inflammation.

The mice were anaesthetized with isoflurane, gently placed on the console, and laid flat on their backs. After gently lifting the tongue, an endoscopy (Karl Storz, Tuttlingen, Germany) was inserted into the mouth with the pharynx exposed. Next, a microsyringe (Hamilton, USA) was used to inject Complete Freund's Adjuvant (CFA, 5 µL or 10 µL, Sigma), a classical inflammatory factor consisting of heat-killed Mycobacterium tuberculosis in non-metabolizable oils (paraffin oil and mannide

monooleate), into the mucosa of the pharynx with an endoscope(5-7). The syringe needles were left in place for 3 min before slowly withdrawing when the injection was completed. After confirming that there was no bleeding under the endoscope, the mice were resuscitated on a heated blanket. The control mice were subjected to the same operation but were injected with saline (10 μL). For pharmacological manipulation, the conventional anti-inflammation agent dexamethasone (DXM, 2 mg/kg) was administered intraperitoneally for 7 consecutive days (from day 0 to day 7 after CFA injection).

Macroscopic observation.
Gross morphological changes in the pharynx of the mice were observed by endoscopic examination. After macroscopic observation of the pharynx, endoscopic scoring was performed to assess the severity of pharyngeal inflammation in the mice (*i.e.*, color, secretion, congestion, and other pathological phenomena) ranging from 0 to 3, with higher scores indicating greater inflammation.

Food intake monitoring
To monitor food intake, individual isolated mice were acclimated to the home cage (25 cm long, 15 cm wide, 10 cm high) for at least 5 days with food and water available ad libitum and rhythmic light. Adapted mice were then given pharyngeal injection of CFA or saline and returned to their home cage after surgery. Food intake was measured daily at the same time for each mouse.

Histological examination.
The mice were anesthetized with pentobarbital sodium (2% w/v, i.p.) and then intracardially perfused with saline and 4% (w/v) paraformaldehyde (PFA). The pharyngeal mucosa was collected, fixed in 4% (w/v) PFA, embedded in paraffin, sectioned, and stained with hematoxylin and eosin (H&E). H&E sections were used to observe hypertrophy, hemorrhagic spots, and inflammatory cell infiltration in the pharynx(8). For immunohistochemistry staining, formalin-fixed and paraffin-embedded tissue sections were deparaffinized and rehydrated, then rinsed in running water until the sections were clear, followed by antigen retrieval in citrate buffer (pH 6.0). The sections were then incubated in 3% hydrogen peroxide for 10 minutes at room temperature, washed 3 times in phosphate-buffered saline (PBS), blocked for 10 minutes and then incubated overnight at 4°C with primary antibodies to tumor necrosis factor-α (TNF-α) (1:400, rabbit, Abcam) or interleukin (IL)-6 (1:400, rabbit, Abcam). After three washes in PBS, sections were incubated with biotinylated goat anti-polyvalent antibody and streptavidin peroxidase. The signal was visualized by DAB staining and the nuclei were restrained with hematoxylin and finally blued with lithium carbonate solution. Positive staining was browned under the microscope and sections incubated without primary antibody were used as negative controls. Finally, the sections were visualized by using an Olympus BX43 light microscope (Olympus, Japan).

Glossopharyngeal and vagal nerves transection.
The mice were anaesthetized and placed flat on the operating table. An incision was made in the neck to expose the subcutaneous muscle, and the muscle groups were dissected layer-by-layer to locate the glossopharyngeal and vagal nerves(9), which were transected with microscissors before injecting CFA. The opposite side was dissected similarly. To prevent infection, the skin of the mouse was sutured and swabbed with iodophor at the end of the procedure.

NJP superganglia injection.
Mice were anaesthetized and placed in the supine position, and incision was made along the neck and slightly extended to the sides. The submandibular gland and thymus were separated to expose the trachea and carotid artery. The NJP superganglia was dissected with fine forceps(10). Care was taken to protect the carotid artery to avoid bleeding. A total of 500 nL of virus was delivered at a rate of 50 nL min^{-1} to each NJP superganglia using a microsyringe. After leaving the needle in place for 3 minutes, close the wound with sterile surgical sutures and disinfect with iodophor.

Viral injection.
The mice were deeply anesthetized using isoflurane with oxygen (3% for induction; 1.5%-2% for maintenance) and mounted on a stereotaxic frame (RWD, Shenzhen, China). The body temperature of the animals was maintained at 36°C throughout the procedure using a heating pad. After cutting the scalp with scissors, a microsyringe with a glass electrode was used to ensure that

the skull was straight in all planes. The location of the target brain region was identified, and a hand drill was used to gently remove the skull above it, before proceeding to viral injection. The virus was injected into the target areas via a microsyringe connected to an infusion pump (Micro 4, WPI). The pumping speed of the microsyringe was 35 nL min^{-1}, and the injection volume varied from 100 to 300 nL according to the expression strength and titer of the virus. The syringe was retained in place for another 10 min after the injection was completed to avoid virus overflow.

For retrograde tracing from the pharynx, the mice were first anesthetized before exposing the injection site with an endoscope and injecting 10 μL retro-AAV-hSyn-Cre-eGFP-WPRE-pA (retro-AAV-Cre-EGFP, AAV2/R, 5.45 × 10^{12} vg mL^{-1}, BrainVTA) into the pharyngeal mucosa using a syringe. The pipette was left in place for an additional 5 min after injection, before removing slowly. Simultaneously, rAAV-Ef1α-DIO-EGFP-WPRE-pA (AAV-DIO-EGFP, AAV2/9, 1.95 × 10^{12} vg mL^{-1}, BrainVTA) was injected into the NJP superganglia. Using the same method, retro-rAAV-hSyn-mCherry-WPRE-pA (retro-AAV-hSyn-mCherry, AAV2/R, 5.32 × 10^{12} vg mL^{-1}, BrainVTA) was injected into the pharyngeal mucosa, and retro-rAAV-hSyn-eGFP-WPRE-pA (retro-AAV-hSyn-EGFP, retro-AAV2/2, 5.31 × 10^{12} vg mL^{-1}, BrainVTA) was injected into the NTS (AP: -8.1 mm, ML: -0.25 mm, DV: -4.75 mm). The mice were allowed to recover for 21 days before perfusion to detect fluorescent signals.

To trace downstream NTS projections, an rAAV-Ef1α-DIO-hChR2(H134R)-mCherry-WPRE-pA (AAV-DIO-ChR2-mCherry, AAV2/9, 1.63×10^{13} vg mL^{-1}, BrainVTA) was injected into the NTS of *DBH-Cre* mice. After 3 weeks, mCherry expression was detected in the whole brain. To trace collateral projections from the same NTS neurons that project to the vBNST, a retro-AAV-hSyn-Cre-WPRE-pA (retro-AAV-hSyn-Cre, AAV2/R, 6.24 × 10^{12} vg mL^{-1}, BrainVTA) virus was injected into the vBNST of C57 mice, while at the same time, an AAV-DIO-ChR2-mCherry virus was infused into the NTS. Fluorescent signals were detected after 21 days of viral expression.

For anterograde monosynaptic tracing, rAAV-hSyn-Cre-mCherry-WPRE-hGH-pA (AAV-hSyn-Cre-mCherry, AAV2/1, 6. 38 × 10^{12} vg mL^{-1}, BrainVTA) was injected into the NJP superganglia to allow the virus to spread antegrade downstream to express *Cre* in C57 mice. Simultaneously, rAAV-Ef1α-DIO-mCherry-WPRE-pA (AAV-DIO-mCherry, AAV2/9, 6.76 × 10^{12} vg mL^{-1}, Brain Case) was injected into the NTS(11, 12). After 3 weeks, the mice were anesthetized and transcardially perfused, before preparing brain slices for tracing mCherry signals or co-staining with antibodies. The same tracing strategy was performed for the NTS→vBNST circuit (vBNST, AP: -0.75 mm, ML: -1.25 mm, DV: -4.25 mm). AAV-hSyn-EGFP-2A-Cre (AAV-Cre-EGFP, AAV2/1, 6.83 × 10^{12} vg mL^{-1}, BrainVTA) was injected into the NTS, and Cre-dependent AAV-DIO-EGFP was injected into the ipsilateral vBNST.

For retrograde monosynaptic tracing, helper viruses containing rAAV-Ef1α-DIO-RVG-WPRE-pA (AAV-DIO-RVG, AAV2/9, 2×10^{12} vg mL^{-1}) and rAAV-EF1α-DIO-EGFP-T2A-TVA (AAV-DIO-EGFP, AAV2/9, 2×10^{12} vg mL^{-1}; 1:1, 200 nL, Brain Case) were injected into the vBNST of *VgluT2-Cre* mice and *Vgat-Cre* mice. Three weeks later, RV-EnvA-ΔG-DsRed (2 ×10^{8} IFU mL^{-1}, 200 nL, Brain Case) was injected into the vBNST using identical conditions and coordinates. 7 days after the last injection, the mice were transcardially perfused and brain slices were prepared to trace the DsRed signals or perform immunohistochemical analysis.

For chemogenetic manipulation, the rAAV-Ef1α-DIO-hM4D(Gi)-mCherry-WPRE-pA (AAV-DIO-hM4Di-mCherry, AAV2/9, 3.69 × 10^{13} vg mL^{-1}, BrainVTA) and the rAAV-Ef1α-DIO-hM3D(Gq)-mCherry-WPRE-pA (AAV-DIO-hM3Dq-mCherry, AAV2/9, 2.69×10^{13} vg mL^{-1}, BrainVTA) viruses were used, and the rAAV-Ef1α-DIO-mCherry-WPRE-pA (AAV-DIO-mCherry, AAV2/8, 8.93 ×10^{12} vg mL^{-1}, BrainVTA) virus was used as the control. For chemogenetic manipulation of $vBNST^{ENK}$ neurons, an AAV-PENK-hM3Dq-mCherry (AAV2/9, 4.85×10^{12} vg mL^{-1}, BrainVTA) was injected into the vBNST, while AAV-PENK-mCherry (AAV2/9, 3.34×10^{13} vg mL^{-1}, BrainVTA) was injected as a control virus.

For optogenetic manipulation, AAV-Ef1α-DIO-eNpHR-mCherry-WPRE-pA (AAV-DIO-eNpHR-mCherry, AAV2/9, 5.17 × 10^{13} vg mL^{-1}, BrainVTA) was used. For wireless optogenetic manipulation, retro-AAV-hSyn-Cre-mCherry-WPRE-pA (retro-AAV-Cre-mCherry, AAV2/R, 5.50 × 10^{12} vg mL^{-1}, BrainVTA) virus was injected into the NTS to define the connections of the pharynx→NJP→NTS circuit. Additionally, rAAV-hSyn-Con Fon-eNpHR-EYFP (AAV-Con fon-eNpHR-EYFP, AAV2/9, 2.10 × 10^{12} vg mL^{-1}, Brain Case) and retro-AAV-hSyn-FlpO-WPRE-pA (retro-AAV-FlpO, AAV2/R, 5.00 × 10^{12} vg mL^{-1}, Brain Case) viruses were separately injected into the NJP and the pharynx.

For pharynx→NJP→NTS→vBNST manipulation, apart from the above mentioned, RV-FlpO-mCherry (2.00 × 10^8 vg mL^{-1}, Brain Case) and rAAV-Ef1α-fDIO-eNpHR-EYFP-WPRE-pA (AAV-fDIO-eNpHR-EYFP, AAV2/9, 5.77 × 10^{12} vg mL^{-1}, Brain Case) viruses were used.

For *in vivo* microendoscope imaging, rAAV-Ef1α-DIO-GCaMP6m-WPRE-pA (AAV-DIO-GCaMP6m, AAV2/9, 1.63 × 10^{13} vg mL^{-1}, BrainVTA) virus was injected into the NTS of *DBH-Cre* mice. The same strategy was applied for injection into the vBNST of *Vgat-Cre* and *VgluT2-Cre* mice.

For *in vivo* electrophysiological recordings, AAV-hSyn-ChR2-mCherry was injected into the NJP superganglia of C57 mice, and AAV-DIO-ChR2-mCherry virus was injected into the NTS of *DBH-Cre* mice.

For whole-cell patch-clamp recordings, AAV-DIO-ChR2-mCherry and rAAV-VgluT2-EGFP-WPRE-pA (AAV-VgluT2-EGFP, AAV2/9, 6.62 × 10^{12} vg mL^{-1}, BrainVTA) viruses were separately injected into the NTS and vBNST of *DBH-Cre* mice. Additionally, AAV-DIO-ChR2-mCherry and AAV-VgluT2-EGFP viruses were injected into the vBNST of *Vgat-Cre* mice to visualize the $vBNST^{Glu}$ and $vBNST^{GABA}$ neurons.

The mice were transcardially perfused with ice-cold 0.9% saline, followed by 4% PFA, for 4 min each. The images of signal expression were obtained using a confocal microscope (LSM980, Zeiss).

Optogenetic and chemogenetic manipulation.
For optogenetic manipulation with an implanted optical fiber, the mice were placed on a stereotaxic apparatus to identify the location of the target brain region. Subsequently, a hand drill was used to gently remove the upper skull, before implanting the optical fiber. Dental cement was used to ensure that the implant was secured to the skull of the mice. The viral expression was completed after 3 weeks. Subsequently, chronically implantable fibers (diameter: 200 μm, Newdoon) were connected to a laser generator using optic fiber sleeves. After 3 weeks of recovery, the delivery of a 5-min pulse of yellow light (594 nm, 5-8 mW, constant) was controlled using a Master-8 pulse stimulator (A.M.P.I.). An identical stimulus protocol was applied to control mice.

For wireless optogenetic manipulation, the mice were subjected to a cervical incision to locate the bilateral NJP superganglia and the posterior wall of the pharynx. After virus injection, the 400 μm-length needle (80-130 mm thick, with a yellow LED) of the wireless optogenetic device (ThinkerTech) was glued to the bilateral NJP superganglia or the posterior wall of the pharynx with tissue glue. A copper wire connected the needle to a 1 cm diameter copper coil. The copper coil was placed subcutaneously on the neck with the indicator facing outwards. Care was taken to avoid compressing the airway or esophagus with the wire to prevent eating or breathing problems in the mice. After 3 weeks, these devices were wirelessly controlled using a radio frequency (RF) power source to inhibit the NJP neurons or their terminals in the pharynx using yellow light (594 nm, constant).

For chemogenetic manipulation, 3 weeks after viral injection, an intraperitoneal injection of CNO (5 mg per kg, Sigma) was administered 30 min before the behavioral tests.

An identical stimulus protocol was applied to control mice. After the completion of all behavioral tests, the mice were killed to verify the virus injection site and the optical fiber site.

Open field test.
To determine the effect of pharyngeal inflammation on anxiety-like behavior, the mice were individually placed at the center of an open field apparatus (50 cm × 50 cm × 30 cm). The mice were immediately allowed to freely explore the apparatus for 5 min and their movement trajectories were recorded by a video camera. The square area at the center of the apparatus (25 cm × 25 cm) was defined as the center zone, and the time spent in this central area and the number of times the mice entered the central area were analyzed offline using EthoVision XT software (Noldus). Two hours before the test, the mice were pre-placed in the behavioral room to adapt to the environment. After each mouse was tested, the instrument was cleaned with 75% ethanol to remove odor clues.

Elevated plus maze test.
The apparatus was placed 100 cm above the floor, which consisted of a central platform (6 cm × 6 cm) and two open arms (30 cm × 6 cm) orthogonal to two closed arms (30 cm × 6 cm × 20 cm).

The mice were placed on the central platform toward an open arm and allowed to explore the maze for 5 min. The trajectories of the mice were captured with a video camera. Finally, EthoVision XT software (Noldus) was used to analyze the time spent in the open arms and the number of times they entered the open arms.

To calculate "anxiety index" values, data from EPM tests were applied to the following formula:

$$\text{Anxiety index}=1-\left\{\frac{\left(\frac{\text{time spent in the open arms}}{\text{total time on the maze}}\right)+\left(\frac{\text{number of entries to the open arms}}{\text{total activity on the maze}}\right)}{2}\right\}$$

The anxiety index values ranged from 0 to 1, with higher values indicating increased anxiety-like behaviors(13).

Light-dark box test.
The light-dark box consisted of a light chamber and a dark chamber of equal sizes (20 cm × 15 cm × 30 cm). The two chambers were separated by a wall with an open gate (5 cm × 5 cm) to allow the mice to freely explore the two chambers. To test the effect of pharyngeal inflammation on anxiety-like behaviors, the mice were individually placed in a light chamber and allowed to freely explore the apparatus for 15 min. The travel trajectories were video-recorded and analyzed offline using EthoVision XT software (Noldus). The time spent in the light chamber and the number of entries into the light chamber were noted.

Microendoscopic imaging and data processing.
Three weeks after virus injection, the mice were anesthetized with isoflurane and mounted on a stereotaxic frame to identify the location of the target brain region. Next, a hand drill was used to remove the upper skull carefully, and an integrated micro-endoscopic GRIN lens (0.5 mm in diameter × 6 mm in length, Inscopix, #1050-002211) was slowly lowered (100 μm min^{-1}) toward the target areas using a stable stereotaxic holder attachment. The GRIN lens was connected to a data acquisition system for online monitoring of calcium signals. Once the GCaMP6m-expressing neurons were detected, the GRIN lens was secured to the mouse skull with dental cement and the lens was capped for protection.

After embedding the GRIN lens in the target brain area, the mice were reared alone for a week to allow them to recover, before proceeding with the experiment. Before the data were recorded, the mice were allowed to move freely in a rectangular recording chamber, and the head was connected to an integrated miniature fluorescence microscope (Inscopix, USA), but no signals were recorded. Ca^{2+} images were obtained after 30 min of adaptation for 3 consecutive days. Imaging data were acquired at 20 Hz for 17 min, and the mice were allowed to move freely as before. The GRIN lenses implanted in the NTS were modified, briefly, three polyimide tubes were attached to the GRIN lens using UV glue and spaced equally. Tungsten wires were inserted through the tubes and were 400-600 mm longer than the tubes to stabilize the brain(14).

For data analysis, fluorescence videos were processed offline with Inscopix data processing software (version 1.1.6). First, the raw data were 2× temporally down-sampled, and motion was corrected using default settings. Then, the fluorescence signals were normalized by their time-averaged mean (ΔF/F calculation), and the normalized movie was used for semiautomatic extraction of Ca^{2+} fluorescent signals associated with individual cells based on principal and independent component analysis (PCA-ICA). Regions of interest (ROIs) identified by PCA-ICA were visually selected as candidate cells based on ΔF/F and image (cell-morphology). Calcium events in the soma were identified by spike inference and calcium values above an automatically calculated threshold.

***In vivo* multi-tetrode recordings.**
For extracellular recordings, eight movable custom-built tetrode arrays were implanted into the target areas of the mice, including the NTS and the vBNST. The tetrode was composed of four twisted platinum/iridium wires (12.5-μm diameter, California Fine Wire, Grover Beach, CA, USA). A screw-based microdrive scaffold housing the electrodes was firmly mounted onto the skull using dental cement. Before recording, the mice were reared alone for at least 3 days to allow them to recover. The mice implanted with electrodes were placed in a cylindrical box wrapped in copper mesh and were allowed to move freely. The electrodes were lowered in steps of 40 μm to record different neuronal ensembles. Neuronal signals were amplified, filtered at a 300-5000 Hz bandwidth, and stored with Neurostudio software (Neurostudio, China), before exporting to Offline Sorter 4

(Plexon, USA) and Neuroexplorer 4 (Nex Technologies, USA) for offline analysis. For electrophysiology combined with optogenetics, an optrode was constructed by surrounding an optical fiber with tetrode wires, and the tip of the optical fiber was approximately 200 µm above the tetrode tips. For optical identification of neurons, blue light pulses (490 nm, 2-ms duration, 20 Hz) were delivered at the end of each recording session at high frequencies. All recorded light-sensitive neurons were classified as wide-spiking putative glutamatergic cells and GABAergic cells using an unbiased κ-means cluster algorithm based on the following three electrophysiological properties: firing rate, trough to peak duration, and half width.

Whole-cell patch-clamp recordings.

For brain slice preparation, the mice were anesthetized with pentobarbital sodium (2% w/v, i.p.) and subsequently intracardially perfused with ~20 mL of ice-cold oxygenated cutting solution containing 93 mM Nmethyl-d-glucamine (NMDG), 1.2 mM NaH_2PO_4, 2.5 mM KCl, 20 mM HEPES, 30 mM $NaHCO_3$, 2 mM thiourea, 25 mM glucose, 3 mM Na-pyruvate, 5 mM Na-ascorbate, 10 mM $MgSO_4$, 0.5 $CaCl_2$, and 3 mM glutathione (GSH). Next, the mouse brain was carefully removed from the skull and placed into a beaker containing ice-cold oxygenated solution of NMDG. The brain was then glued on the bed plate of a vibratome, and coronal slices (280 µm) that contained the NTS or vBNST were cut in ice-cold cutting solution and sectioned at a rate of 0.18 mm s^{-1} (VT1200s, Leica). These slices were initially incubated in NMDG at 33°C for 10-12 min and subsequently transferred into N-2-hydroxyethylpiperazine-N-2-ethanesulfonic acid (HEPES) artificial cerebrospinal fluid (ACSF), which contained 2.5 mM KCl, 92 mM NaCl, 30 mM $NaHCO_3$, 20 mM HEPES, 1.2 mM NaH_2PO_4, 2 mM thiourea, 25 mM glucose, 3 mM Na-pyruvate, 5 mM Na-ascorbate, 2 mM $MgSO_4$, 2 mM $CaCl_2$, and 3 mM GSH, at 25°C for at least 1 h. When incubation was complete, the slices were placed in a recording chamber (Warner Instruments, USA) for electrophysiological recording and were continuously perfused with oxygenated standard ACSF (2.4 mM $CaCl_2$, 3 mM KCl, 129 mM NaCl, 20 mM $NaHCO_3$, 1.3 mM $MgSO_4$, 1.2 mM KH_2PO_4, and 10 mM glucose) at a rate of 2.5-3 mL min^{-1} at 32°C that was maintained using an in-line solution heater (TC-344B, Warner Instruments). The pH of all ACSFs was set to 7.3-7.4, and the osmolarity was adjusted to 300-305 mOsm kg^{-1}. During slice preparation and electrophysiology recording, all solutions were continuously bubbled with 95% O_2/5% CO_2.

Whole-cell patch-clamp recordings were performed on visualized NTS and vBNST neurons using an ×40 water immersion lens (BX51WI, Olympus) and an infrared-sensitive charge-coupled device (CCD) camera. Patch pipettes (3-5 MΩ) were pulled from borosilicate glass capillaries (VitalSense Scientific Instruments Co., Ltd) using a four-stage horizontal micropipette puller (P1000, Sutter Instruments). Glass pipettes filled with intracellular solution containing 10 mM HEPES, 5 mM KCl, 130 mM K-gluconate, 0.6 mM EGTA, 2 mM $MgCl_2$, 2 mM Mg-ATP, and 0.3 mM Na-GTP (osmolarity: 285-290 mOsm kg^{-1}, pH: 7.2) were used for voltage clamp recording. Signals were amplified with a Multiclamp 700B amplifier, low-pass filtered at 2.8 kHz, digitized at 10 kHz, and recorded in a computer for offline analysis using Clampfit 10.7 software (Molecular Devices).

For light-evoked response, optical stimulation was performed using a laser (Shanghai Fiblaser Technology Co., Ltd. China) through an optical fiber (diameter: 200 µm, Newdoon, Hangzhou) positioned 0.2 mm above the surface of the target brain region. To verify the functional characteristics of the AAV-DIO-ChR2-mCherry virus, mCherry-labelled neurons that expressed ChR2 in the NTS and vBNST were visualized and subjected to blue laser light (473 nm, 5-10 mV) using 5-Hz and 10-Hz stimulation protocols with a pulse width of 15 ms. Light-induced excitatory postsynaptic potential (EPSP) and inhibitory postsynaptic potential (IPSP) were recorded in the $vBNST^{GABA}$ and $vBNST^{Glu}$ neurons after photostimulation of ChR2-expressing NTS^{NE} fibers in the vBNST slices. Additionally, light-evoked inhibitory postsynaptic currents were recorded at -70 mV after photostimulation of ChR2-expressing $vBNST^{GABA}$ fibers in the $vBNST^{Glu}$ slices.

Immunofluorescent staining.

After being deeply anesthetized with pentobarbital sodium (2% w/v, i.p.), the mice were perfused intracardially with saline and 4% (w/v) PFA. The brain was removed and soaked in 4% (w/v) PFA to fix. Gradient dehydration was conducted in 20% (w/v) and 30% (w/v) sucrose, before generating coronal frozen sections (10-40 µm thick). The sections were washed three times with PBS on a shaker for further immunoreactive staining. Next, the sections were blocked in 5% (w/v) donkey serum and 0.5% (v/v) Triton X-100 for 1 h at room temperature. Following blocking, the sections

were incubated with primary antibodies diluted with blocking solution, including anti-c-Fos (1:500, rabbit, Synaptic Systems; 1:500, mouse, Abcam), anti-glutamate (1:500, rabbit, Sigma; 1:500, mouse, Sigma), anti-GABA (1:500, rabbit, Sigma; 1:500, mouse, Sigma), and anti-DBH (1:500, mouse, Santa Cruz), anti-NPY (1:500, rabbit, Abcam), anti-CRF (1:200,rabbit, Proteintech), anti-NOC (1:100, rabbit, Affinity Bioscience), anti-PENK (1:200, rabbit, Abclonal) at 4°C for 24-48 h. Before incubation with secondary antibodies, the sections were washed with PBS three times. Then, the sections and corresponding fluorophore labelled secondary antibodies (1:500, Invitrogen) were diluted with blocking solution at room temperature for 1.5 h, followed by staining with 4-diamino-2-phenylindole (DAPI; 1:1000, Sigma) for 3 min. Finally, the sections were mounted on coverslips after adding antifade mounting media (Vectashield Vibrance), and fluorescence signals were visualized using a Zeiss LSM980 microscope. For the number of c-Fos positive cells, 40× magnification was used for imaging; two slices randomly picked per mouse were imaged. Further analysis, such as those of cell counts and colocalization, were conducted using Image J software (Fiji edition, NIH). The intensity of colocalization was adjusted to accurately count moderately to highly immunoreactive cells. A mean count per mouse was used for statistical evaluation.

Quantification and statistical analysis.

The Shapiro-Wilk test was used to check the normality of data. A paired or unpaired two-tailed Student's *t* test was conducted for the statistical comparisons of data between two groups. Nonparametric Mann-Whitney U test was performed if data were not normally distributed. For some data, Pearson's correlation test was used. For experimental groups with multiple comparisons, data were analyzed using one-way and two-way analysis of variance (ANOVA) and post-hoc analyses. The sample sizes in our study were not predetermined by any statistical methods but were similar to those reported in previous publications. All data in this study are presented as the mean ± SEM The significance levels are indicated as $^{*}P < 0.05$, $^{**}P < 0.01$, and $^{***}P < 0.001$. n.s., not significant. GraphPad Prism 8 (Graph Pad Software, Inc.) was used for statistical analysis and graphing.

Data availability.

All necessary data have been included in the supporting information.

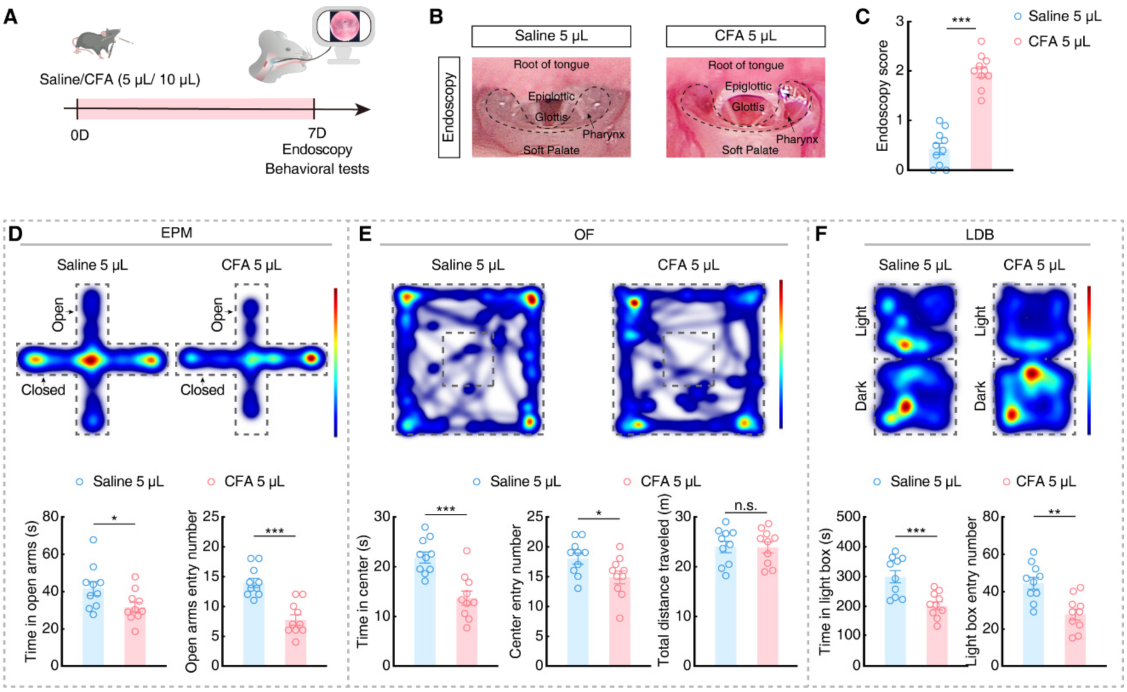

Fig. S1. Endoscopy scores and anxiety-like behaviors in mice at 5 µL of CFA.

(*A*) Schematic of developing a murine model of pharyngeal inflammation by Complete Freund's Adjuvant (CFA) injection.

(*B*) Representative endoscopic images showing the pharynx in saline or CFA 5 µL mice. The dashed box and the long arrow (➛) indicate the pharynx, and the short arrow (➤) indicates pharyngeal congestion and secretion.

(*C*) Schematic of the injection of saline or CFA 5 µL in mice and the endoscopy score of injected mice (*n* = 10 mice per group; t_{18} =9.948, $P < 0.0001$).

(*D* to *F*) Representative heatmaps of the travel trajectory and summarized data for the EPM (*D*), OF (*E*) and LDB (*F*) tests with saline or CFA 5 µL mice (*n* = 10 mice per group; *D*, left, t_{18} = 2.184, P = 0.0425; right, t_{18} = 5.330, $P < 0.0001$; *E*, left , t_{18} = 4.511, P = 0.0003; middle, t_{18} = 2.265, P = 0.0361; right, t_{18} = 0.04788, P = 0.9623; *F*, left, t_{18} = 3.945, P = 0.0009; right, t_{18} = 3.890, P = 0.0011).

Significance was assessed by two-tailed unpaired Student's *t* tests in (*D, E* and *F*). All data are presented as the mean ± SEM. $^{*}P < 0.05$; $^{**}P < 0.01$; $^{***}P < 0.001$, n.s., not significant.

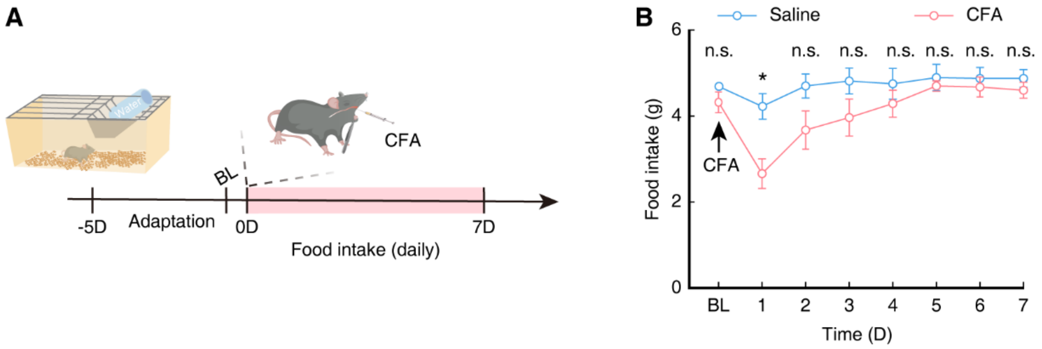

Fig. S2. Food intake in mice.

(*A*) Schematic for observation of food intake behaviors in mice with CFA-induced pharyngitis. BL, baseline.

(*B*) Food intake during each 24h period for 7 days following injection of CFA or saline in pharyngitis or control mice (saline, n = 7 mice; CFA, n = 8 mice, $F_{1,13}$ = 9.164; BL, $P > 0.9999$; D1, $P = 0.0351$; D2, $P = 0.6108$; D3, $P > 0.9999$; D4, $P > 0.9999$; D5, $P > 0.9999$; D6, $P > 0.9999$; D7, $P > 0.9999$).

Significance was assessed by two-way repeated-measures analysis of variance (ANOVA) with post-hoc comparisons between groups in (*B*). All data are presented as the mean ± SEM. $^*P < 0.05$, n.s., no significant.

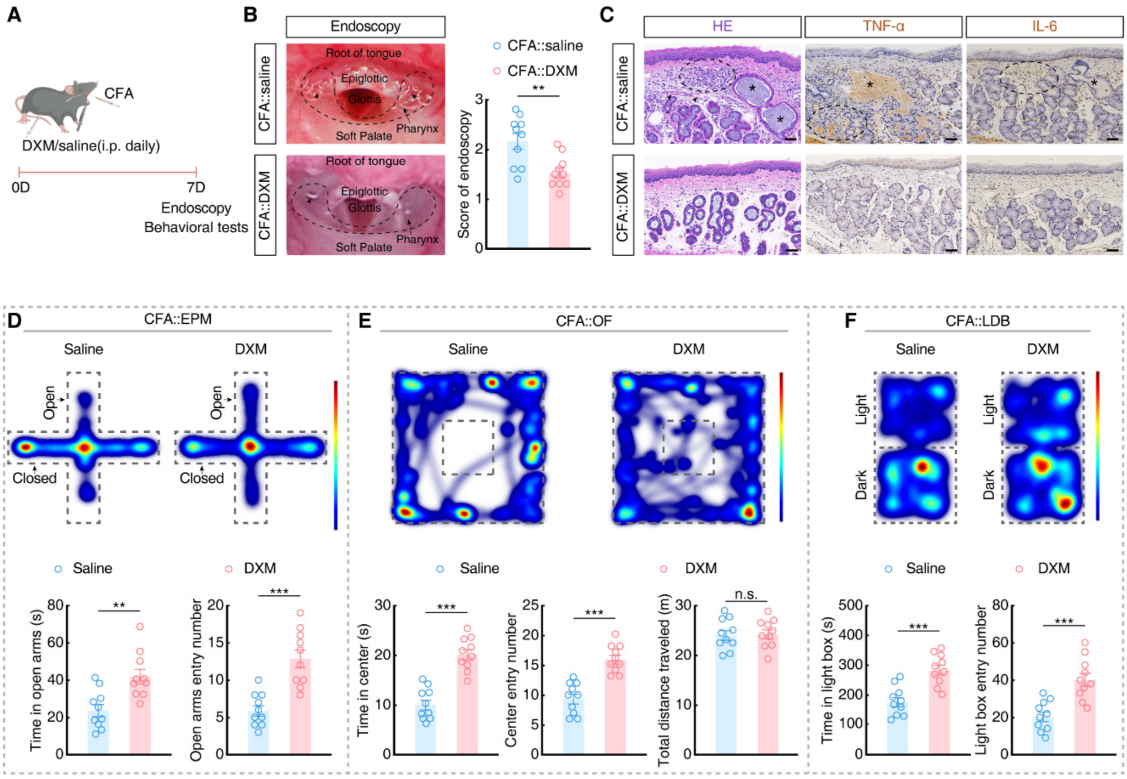

Fig. S3. Dexamethasone can alleviate pharyngeal inflammation and anxiety-like behaviors in CFA mice.

(*A*) Schematic of the intraperitoneal injection (i.p.) of dexamethasone (DXM) in CFA mice.

(*B*) Representative endoscopic images showing the pharynx in saline and CFA mice. The dashed box and the long arrow (➛) indicate the pharynx, and the short arrow (➤) indicates pharyngeal congestion and secretion (left). Endoscopic scoring of CFA mice injected with saline or DXM (right; *n* = 10 mice per group; t_{18} = 3.416, *P* = 0.0031).

(*C*) Representative images of H&E staining and inflammatory cytokine (IL-6, TNF-α) staining of the pharynx. Hemorrhagic patches (➤), retention of secretion in mucus glands (✱), and infiltration of inflammatory cells or factors (dashed box) were improved in CFA mice injected with DXM compared to those injected with saline. Scale bars, 50 μm.

(*D* to *F*) Representative heatmaps of the travel trajectory and summarized data of CFA mice injected with saline or DXM in the EPM (*D*), OF (*E*), and LDB (*F*) tests (*n* = 10 mice per group; *D*, left, t_{18} = 3.186, *P* = 0.0051, right, t_{18} = 5.093, *P* < 0.0001; *E*, left , t_{18} = 6.926, *P* < 0.0001, middle , t_{18} = 5.805, *P* < 0.0001, right, t_{18} = 0.1659, *P* = 0.8701; *F*, left, t_{18} = 4.725, *P* = 0.0002, right, t_{18} = 4.517, *P* = 0.0003).

Significance was assessed by two-tailed unpaired Student's *t* tests in (*B*, *D*, *E* and *F*). All data are presented as the mean ± SEM. ***P* < 0.01, ****P* < 0.001, n.s., not significant.

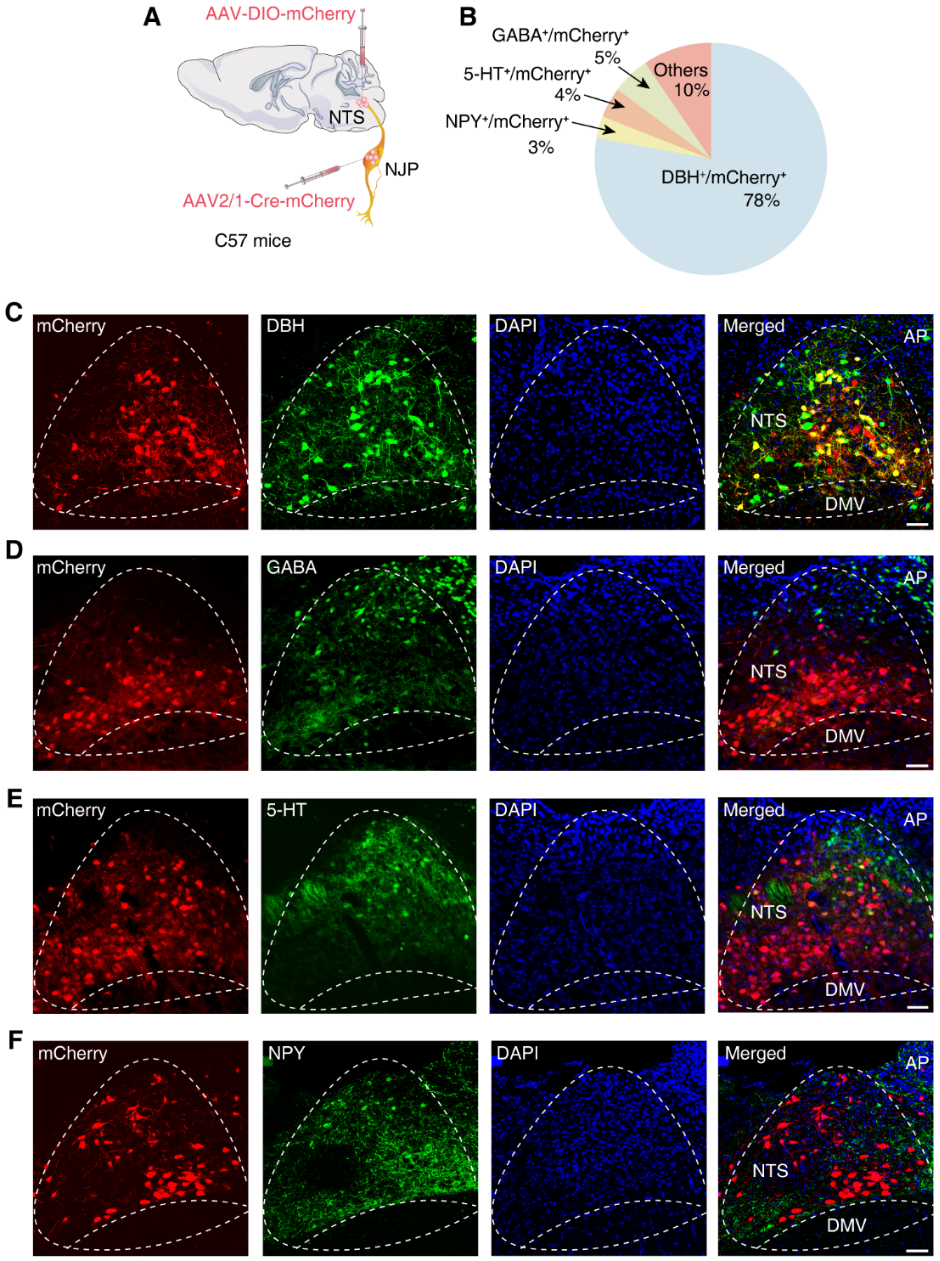

Fig. S4. The NTS neurons receiving NJP projection are colocalized with neuronal markers.

(*A*) Schematic of the viral injection.

(*B*) Proportion of $mCherry^+$ neurons colocalized with neuronal markers ($n = 5$ slices from 5 mice).

(*C* to *F*) $mCherry^+$ neurons in the NTS colocalized with DBH antibody (*C*), GABA antibody (*D*), 5-HT antibody (*E*), and NPY antibody (*F*). Scale bars, 50 μm.

DBH, dopamine β-hydroxylase. GABA, γ-aminobutyric acid. 5-HT, 5-hydroxytryptamine. NPY, neuropeptide Y.

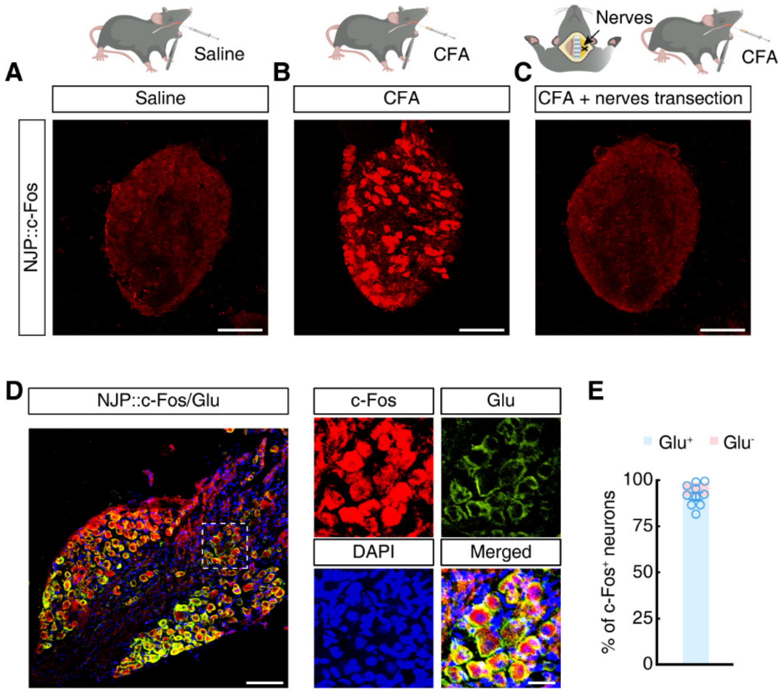

Fig. S5. Enhanced excitability of NJP neurons in CFA mice.

(*A* to *C*) Representative images showing c-Fos-positive neurons in the NJP superganglia of saline (*A*), CFA (*B*), and CFA with glossopharyngeal and vagal nerves transection (*C*) mice. Scale bars, 100 μm.

(*D*) Representative images of c-Fos-positive neurons in the NJP neurons (left) and colocalized with a glutamate antibody (right). Scale bars, 100 μm (left) or 20 μm (right).

(*E*) Statistiscal data showing that c-Fos-labelled neurons in the NJP superganglia of CFA mice predominantly colocalized with a glutamate antibody (n = 10 slices from 5 mice).

All data are presented as the mean ± SEM.

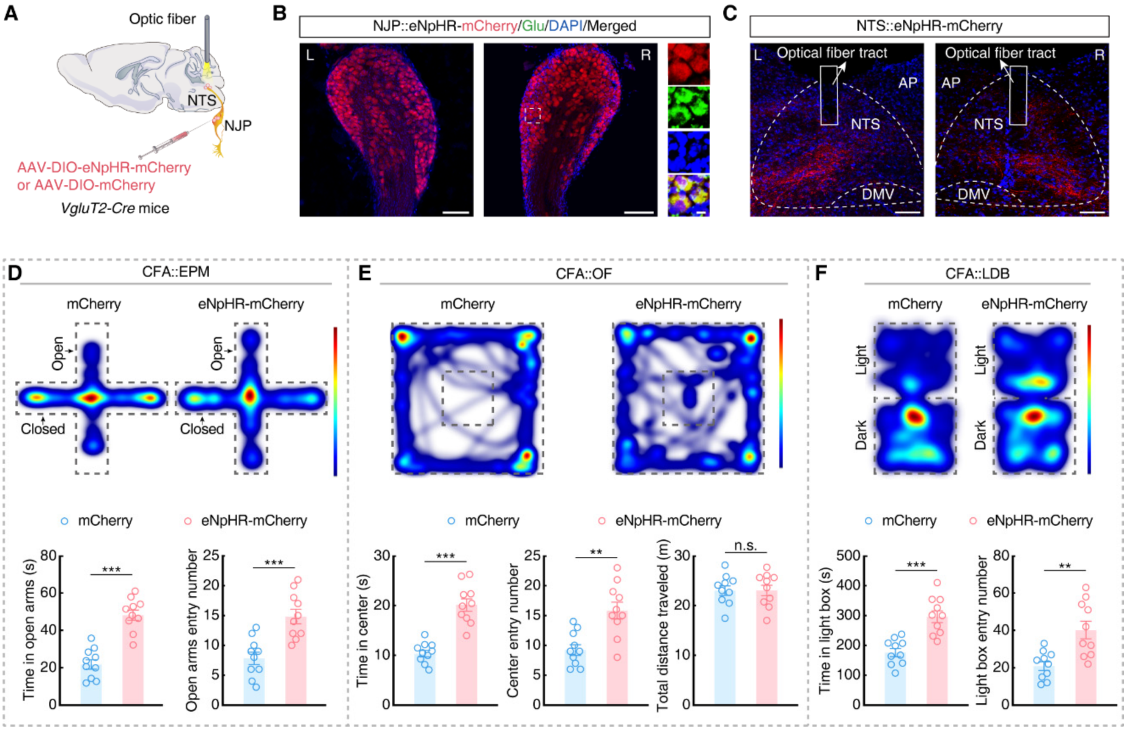

Fig. S6. Inhibition of NJP→NTS circuit alleviates pharyngeal inflammation-induced anxiety-like behaviors.

(*A*) Schematic of the optogenetic inhibition of the NJP→NTS circuit.

(*B*) Representative images of the infusion site and mCherry⁺ neurons (detected with glutamate antibody) within NJP superganglia. Scale bars, 100 μm. White dashed boxes show areas of the NJP superganglia magnified in single channel views at the right. Scale bars, 10 μm. L: left; R: right.

(*C*) Representative images of the projection fibers and optical fiber placement in the NTS. Scale bars, 100 μm. L: left; R: right.

(*D* to *F*) Representative heatmaps of the travel trajectory and summarized data for the EPM (*D*), OF (*E*), and LDB (*F*) tests with mCherry and eNpHR-mCherry CFA mice. (n = 10 per group; *D*, left, t_{18} = 6.999, P < 0.0001; right; t_{18} = 4.379, P = 0.0004; *E,* left, t_{18} = 6.907, P < 0.0001; middle, t_{18} = 3.926, P = 0.0010; right, t_{18} = 0.01108, P = 0.9913; *F,* left, t_{18} = 5.034, P < 0.0001; right, t_{18} = 3.684, P = 0.0017).

Significance was assessed by two-tailed unpaired Student's t tests in (*D*, *E* and *F*). All data are presented as the mean ± SEM. **P < 0.01, ***P < 0.001, n.s., not significant.

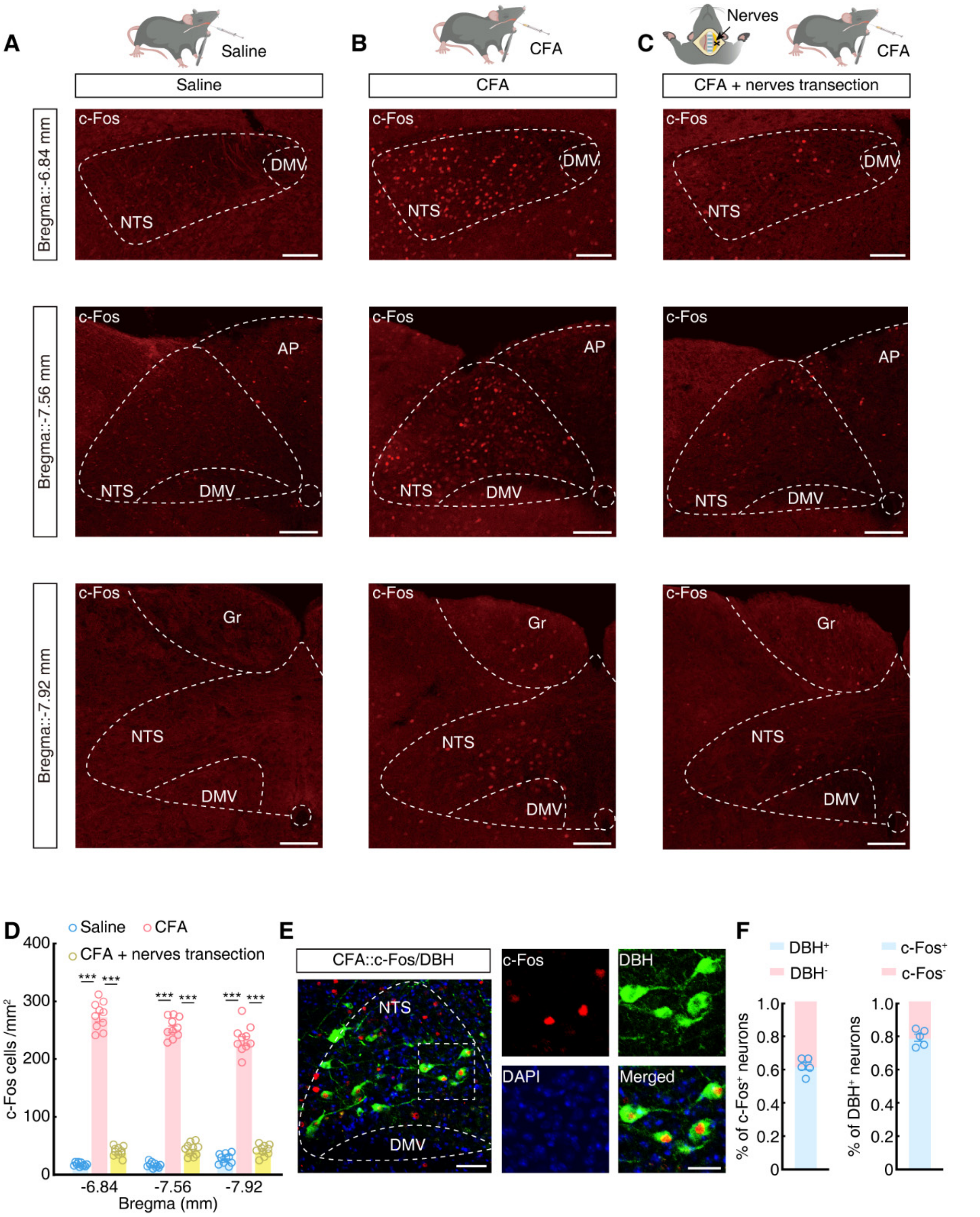

Fig. S7. Enhanced excitability of NTS^{NE} neurons in CFA mice.

(*A* to *C*) Representative images showing c-Fos-positive neurons in the NTS of mice treated with saline (*A*), CFA (*B*), and CFA with glossopharyngeal and vagal nerves transection (*C*). Scale bars, 100 µm.

(*D*) Summarized data on c-Fos-positive neurons (n = 10 slices per group from 5 mice; Bregma = -6.84 mm, $F_{2,\,27}$ = 964.0, saline vs CFA, P < 0.0001, CFA vs CFA + nerves transection, P < 0.0001; Bregma = -7.56 mm, $F_{2,\,27}$ = 1126, saline vs CFA, P < 0.0001, CFA vs CFA + nerves transection,

$P < 0.0001$; Bregma = -7.92 mm, $F_{2, 27} = 544.8$, saline vs CFA, $P < 0.0001$, CFA vs CFA + nerves transection, $P < 0.0001$).

(*E*) Representative images showing c-Fos-positive neurons in the NTS (left) and colocalized with DBH antibody (right). Scale bars, 50 μm (left) or 20 μm (right).

(F) Quantitative analysis showing ~62% of c-Fos^+ neurons colocalized with DBH antibody and ~79% of DBH^+ neurons colocalized with c-Fos antibody in CFA mice.

Significance was assessed by one-way RM ANOVA with Bonferroni post-hoc analysis in (*D*). All data are presented as the mean ± SEM. ***$P < 0.001$.

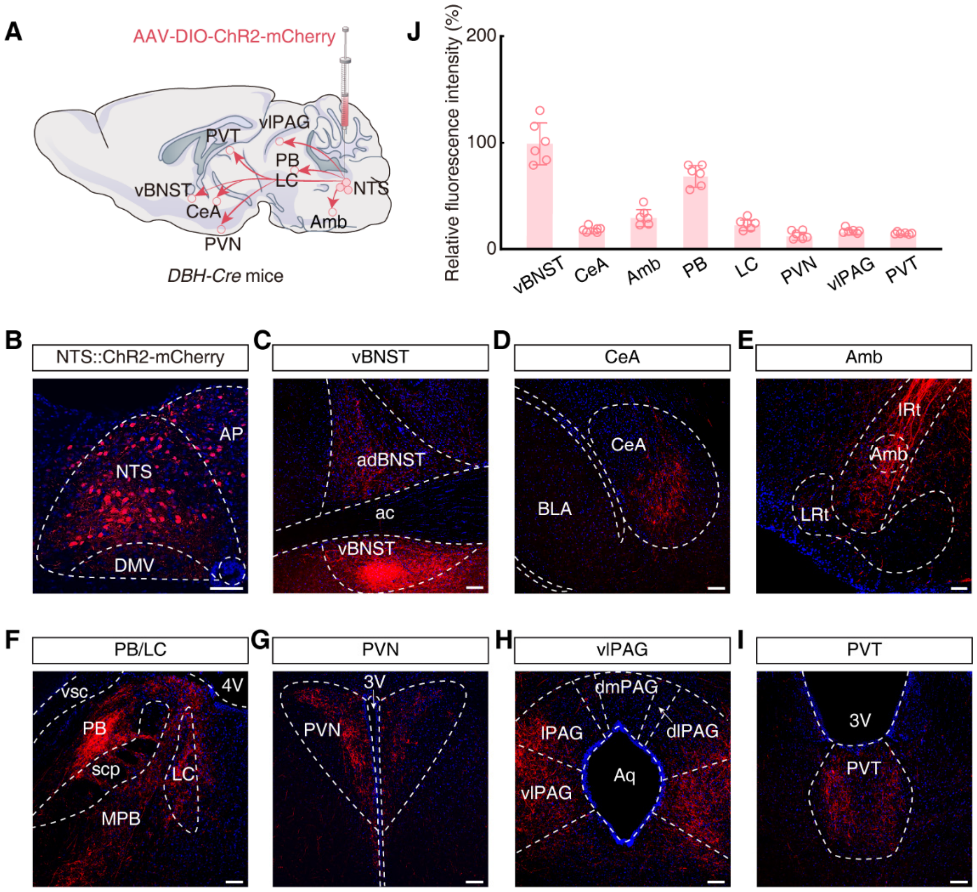

Fig. S8. Outputs of NTS^{NE} neurons.

(*A*) Schematic of the viral injection.

(*B*) Representative images of mCherry^{+} neurons in the NTS of *DBH-Cre* mice. Scale bar, 100 µm.

(*C* to *I*) Representative images of mCherry signals in the vBNST, CeA, Amb, PB, LC, PVN, vlPAG, and PVT of *DBH-Cre* mice injected with AAV-DIO-ChR2-mCherry in the NTS. Scale bars, 100 µm.

(*J*) Summarized data of the relative fluorescence intensities in the indicated regions ($n = 5$ slices per group from 5 mice).

CeA, the central nucleus of the amygdala. Amb, ambiguus nucleus. PB, parabrachial nucleus. LC, locus coeruleus. PVN, paraventricular hypothalamic nucleus. vlPAG, ventrolateral periaqueductal gray. PVT, paraventricular thalamic nucleus. All data are presented as the mean ± s.e.m.

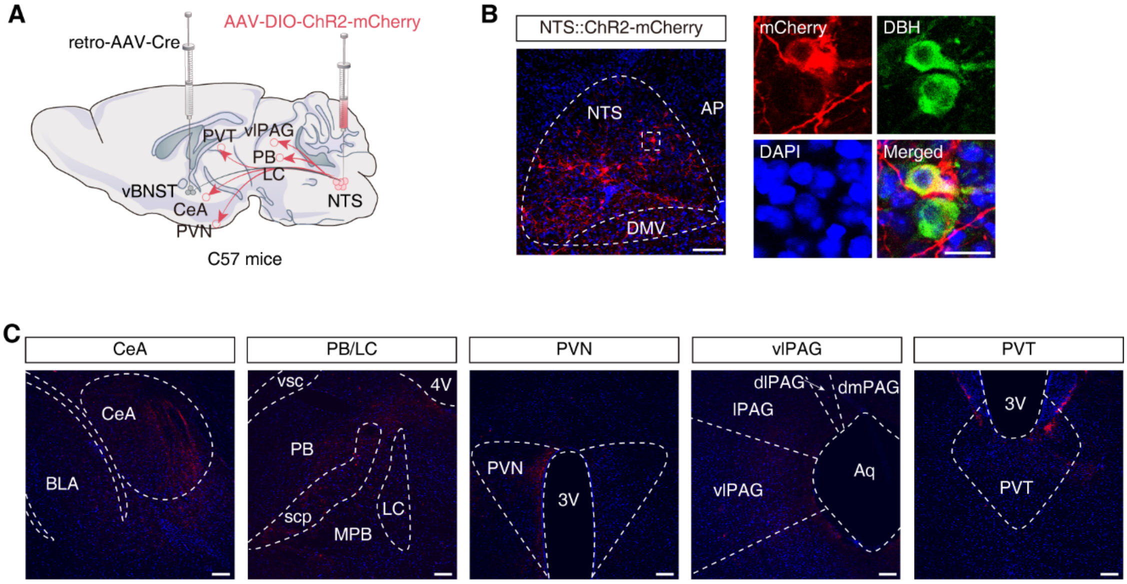

Fig. S9. Collateral projections from NTS neurons that project to vBNST.

(*A*) Schematic for viral injection of retro-AAV-Cre into the vBNST and AAV-DIO-ChR2-mCherry into the NTS.

(*B*) Representative images of mCherry$^+$ neurons in the NTS (left), colocalized with DBH antibody (right). Scale bars, 100 μm or 20 μm.

(*C*) Representative images of mCherry signals in the CeA, PB, LC, PVN, vlPAG, and PVT. Scale bars, 100 μm.

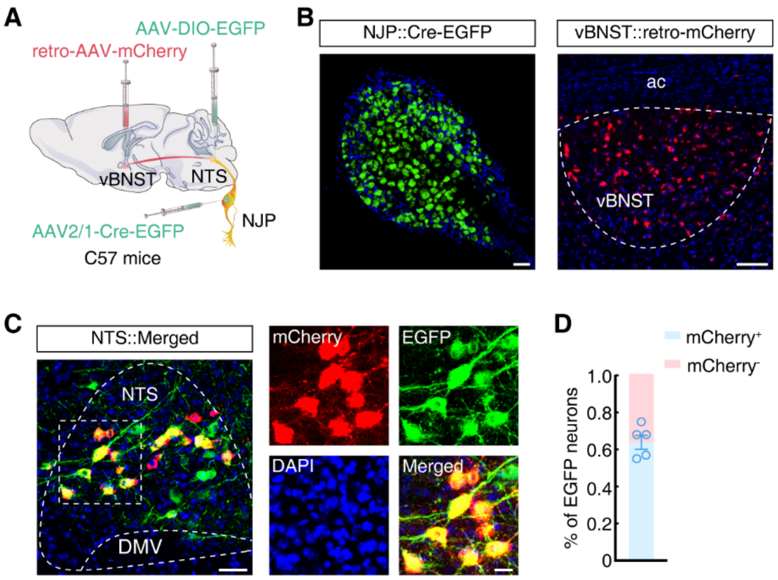

Fig. S10. Characterization of an NJP→NTS→vBNST circuit.

(*A*) Schematic of viral injections for the NJP→NTS→vBNST triple tracing strategy.

(*B*) Representative images of EGFP^{+} neurons in the NJP and the infusion site in the vBNST. Scale bars, 50 μm or 100 μm.

(*C* and *D*) Representative images and quantitative analysis of ~65% EGFP signals colocalization with mCherry signal in the NTS. Scale bar, 100 μm or 20 μm.

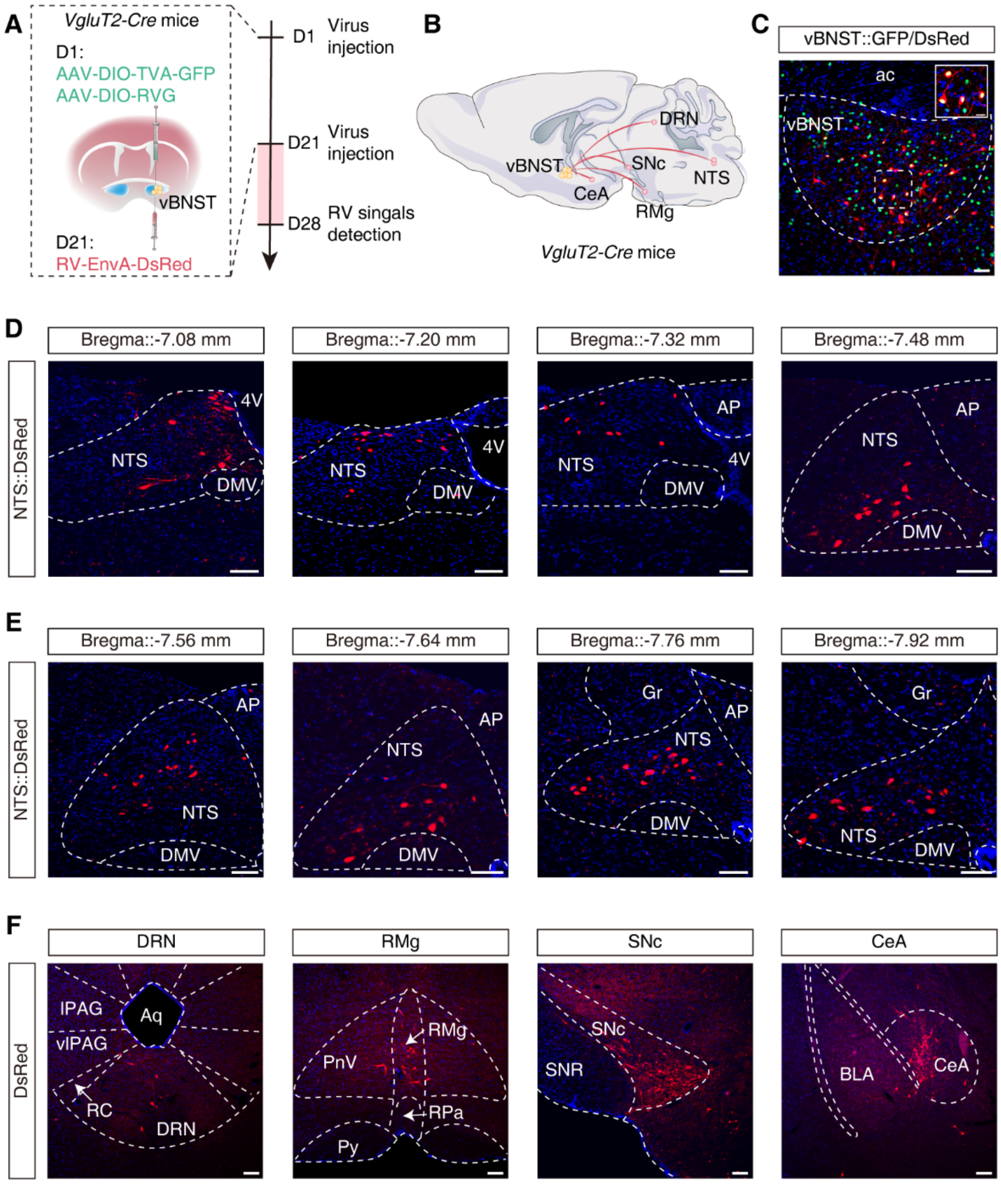

Fig. S11. Retrograde viral tracing of vBNST^{Glu} inputs.

(*A*) Schematic for cell type-specific retrograde trans-monosynaptic tracing of projections to the vBNST.

(*B*) Mapping inputs of vBNST^{Glu} neurons.

(*C*) Representative images showing the infusion site within the vBNST of *VgluT2-Cre* mice. Starter cells (yellow) co-express AAV-DIO-TVA-GFP, AAV-DIO-RVG (green) and RV-EnvA-ΔG-DsRed (red). Scale bar, 50 μm. The white box depicts the area shown in the box of the vBNST. Scale bar, 20 μm.

(*D* and *E*) Representative images of DsRed signals in different bregma sites in the NTS. Scale bars, 100 μm.

(*F*) Representative images of DsRed signals in the DRN, RMg, SNc, and CeA of *VgluT2-Cre* mice. Scale bars, 100 μm.

DRN, dorsal raphe nucleus. RMg, raphe magnus nucleus. SNc, substantia nigra, compact part.

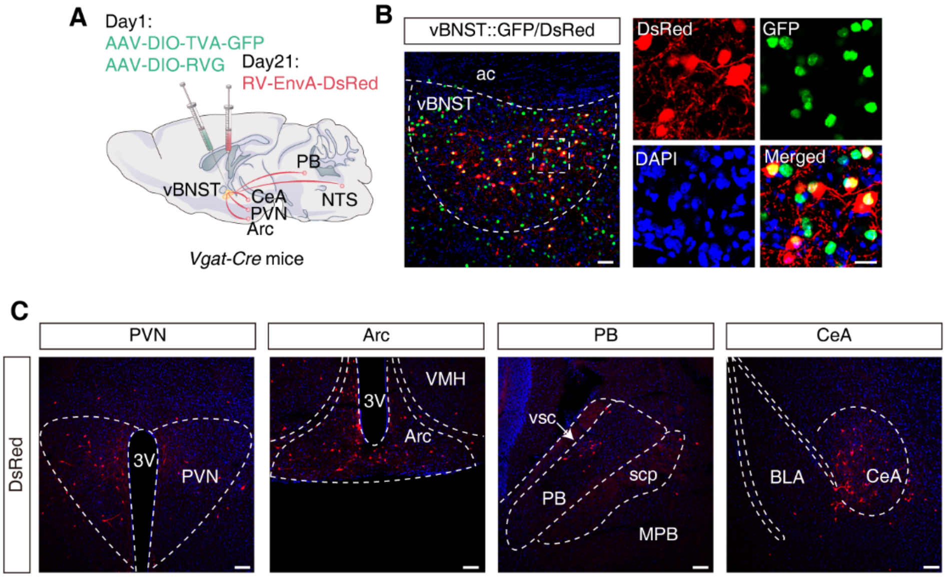

Fig. S12. Mapping inputs of vBNST^{GABA} neurons.

(*A*) Schematic of the viral injection.

(*B*) Typical images showing the infusion site within the vBNST. Scale bars, 50 μm (left) or 20 μm (right).

(*C*) Representative images of DsRed signals in the PVN, Arc, PB, and CeA of *Vgat-Cre* mice. Scale bars, 100 μm.

Arc, arcuate hypothalamic nucleus.

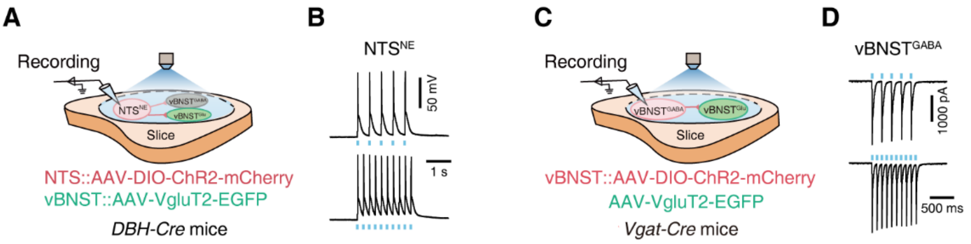

Fig. S13. Recording configuration in acute slices.

(*A*) Schematic of the viral injection and recording configuration in acute slices.

(*B*) Sample traces of action potentials recorded from NTS^{NE} neurons.

(*C*) Schematic of the viral injection and recording configuration in acute slices.

(*D*) Sample traces of action currents recorded from $vBNST^{GABA}$ neurons.

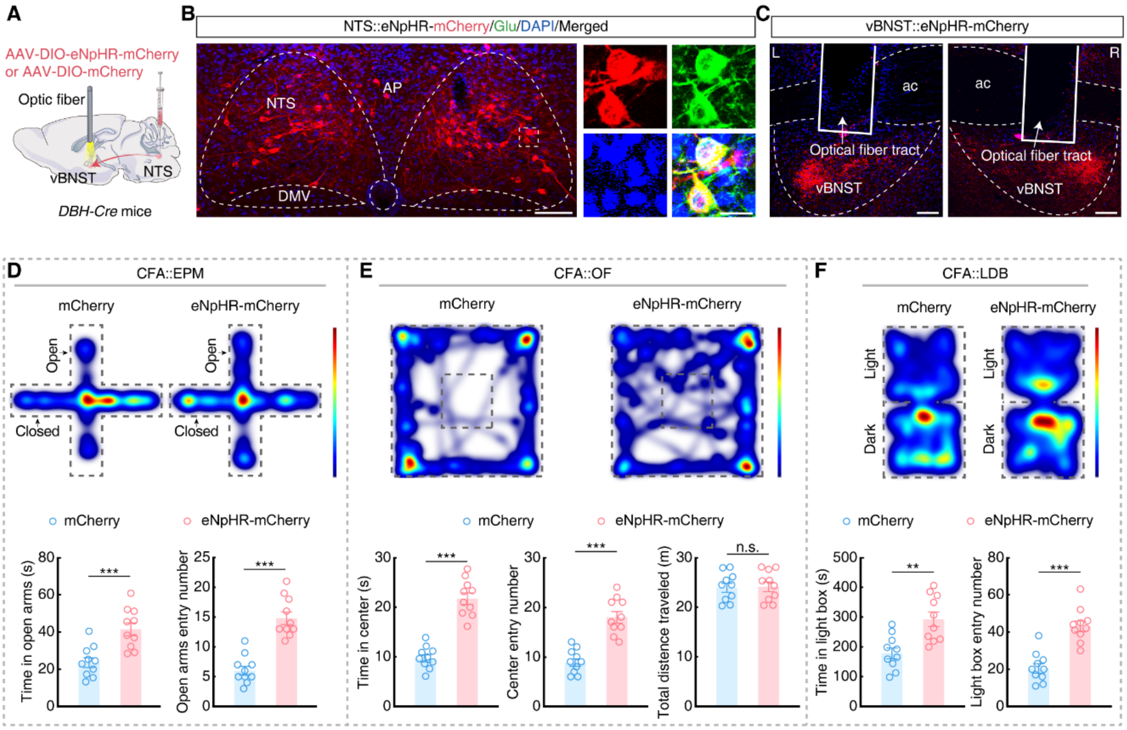

Fig. S14. Inhibition of the NTS^{NE}→vBNST circuit alleviates pharyngeal inflammation-induced anxiety-like behaviors.

(*A*) Schematic for optogenetic inhibition of the NTS^{NE}→vBNST circuit.

(*B*) Representative images of mCherry signals in the NTS. Scale bar, 100 μm. White dashed box indicates region magnified in single channel views at right. Scale bar, 20 μm.

(*C*) Representative images of mCherry^{+} projections and optical fiber placement in the vBNST. Scale bars, 100 μm. L: left; R: right.

(*D* to *F*) Representative heatmaps of the travel trajectory and summarized data for the EPM (*D*), OF (*E*), and LDB (*F*) tests of CFA mice expressing eNpHR-mCherry or the mCherry control virus. (n = 10 mice per group; *D*, left, t_{18} =4.145, P = 0.0006; right, t_{18} = 6.780, P < 0.0001; *E*, left, t_{18} = 8.791, P < 0.0001; middle, t_{18} = 6.573, P < 0.0001; right, t_{18} = 0.1730, P = 0.8646; *F,* left, t_{18} = 3.757, P = 0.0014; right, t_{18} = 5.847, P < 0.0001).

Significance was assessed by two-tailed unpaired Student's *t* tests in (*D*, *E* and *F*). All data are presented as the mean ± SEM. **P < 0.01, ***P < 0.001, n.s., not significant.

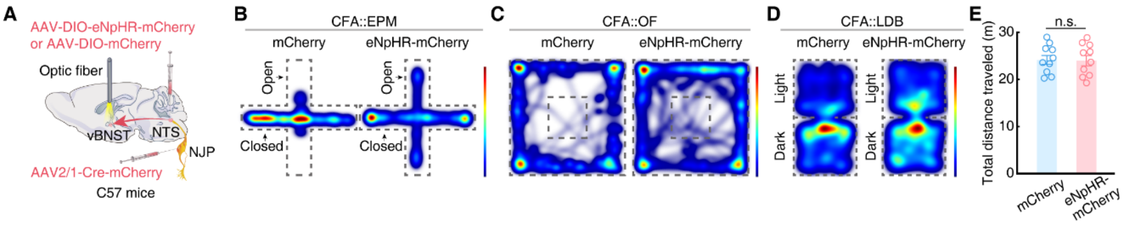

Fig. S15. Inhibition of the NJP→NTS→vBNST circuit alleviates pharyngeal inflammation-induced anxiety-like behaviors.

(*A*) Schematic of the viral injection.

(*B* to *D*) Representative heatmaps of travel trajectory from mCherry or eNpHR-mCherry CFA mice in the EPM (*B*), OF (*C*), and LDB (*D*) tests.

(*E*) Summarized data for the total distance traveled of OF test from mCherry or eNpHR-mCherry CFA mice ($n = 10$ mice per group; $t_{18} = 0.1054$, $P = 0.9172$).

Significance was assessed by two-tailed unpaired Student's *t* test in (*E*). All data are presented as the mean ± SEM. n.s., not significant.

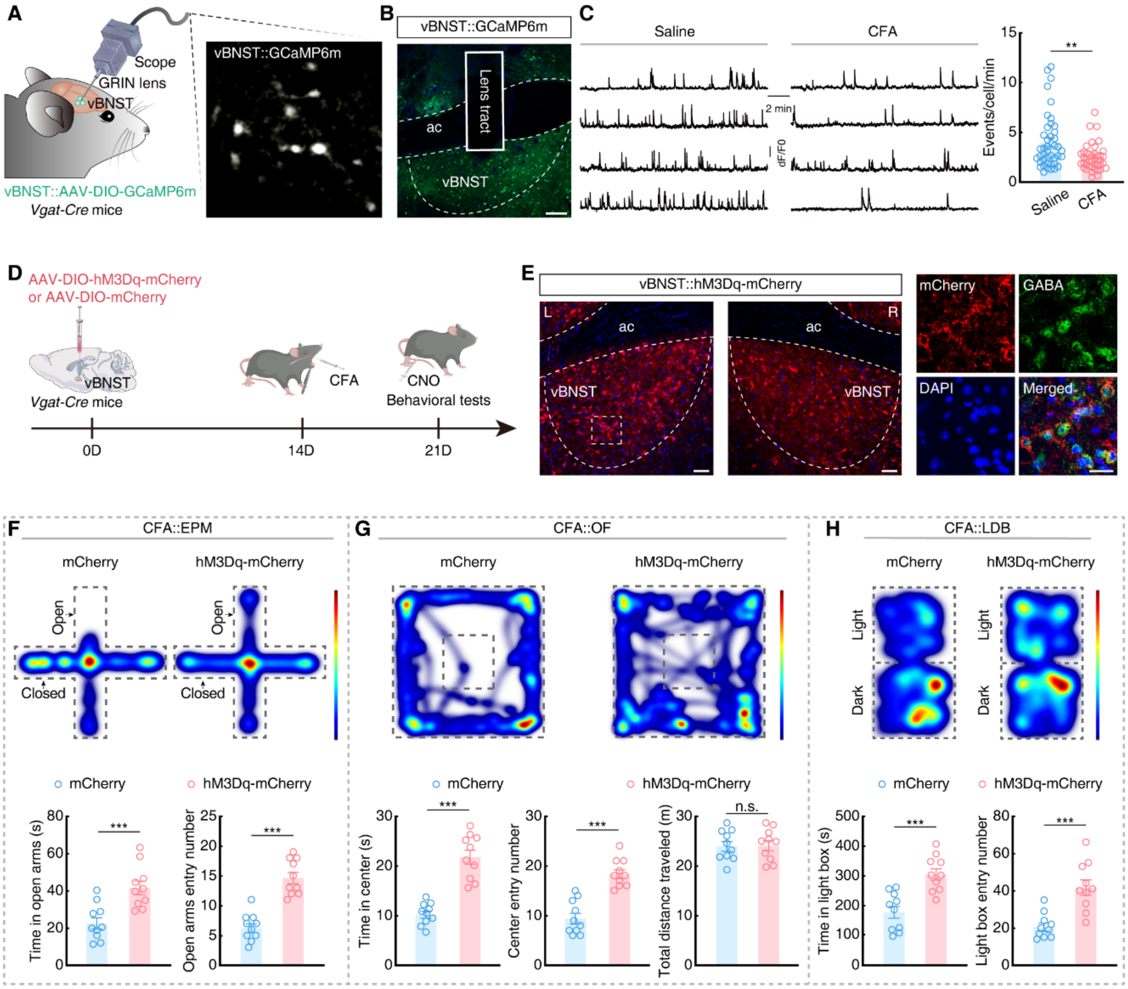

Fig. S16. Activation of vBNST^{GABA} neurons alleviates pharyngeal inflammation-induced anxiety-like behaviors.

(*A*) Schematic of the viral injection and microendoscopic imaging in freely moving mice.

(*B*) Representative image showing the expression of GCaMP6m and the GRIN lens tract in the vBNST. Scale bar, 100 μm.

(*C*) Sample traces (left) and summarized data (right) showing the spontaneous Ca^{2+} transient frequency of vBNST^{GABA} neurons from saline and CFA mice (Saline, n = 42 cells; CFA, n = 42 cells; U = 573.5, P = 0.0084).

(*D*) Schematic of viral injection and chemogenetic activation design.

(*E*) Representative images showing the expression of hM3Dq-mCherry in the vBNST (left) and colocalized with GABA antibody (right). Scale bars, 50 μm (left) or 20 μm (right). L: left; R: right.

(*F* to *H*) Representative heatmaps of the travel trajectory and summarized data of mCherry and hM3Dq-mCherry CFA mice in the EPM (*F*), OF (*G*), and LDB (*H*) tests (after injection of CNO, n = 10 mice per group; *F*, left, t_{18} = 3.987, P = 0.0009, right, t_{18} = 7.044, P < 0.0001; *G*, left, t_{18} = 7.350, P < 0.0001, middle, t_{18} = 6.384, P < 0.0001, right (m), t_{18} = 0.1001, P = 0.9213; *H*, left , t_{18} = 4.586, P = 0.0002, right , t_{18} = 4.574, P = 0.0002).

Significance was assessed by Mann-Whitney test in (*C*), and two-tailed unpaired Student's *t* tests in (*F*, *G* and *H*). All data are presented as the mean ± SEM. $^{**}P < 0.01$, $^{***}P < 0.001$. n.s., not significant.

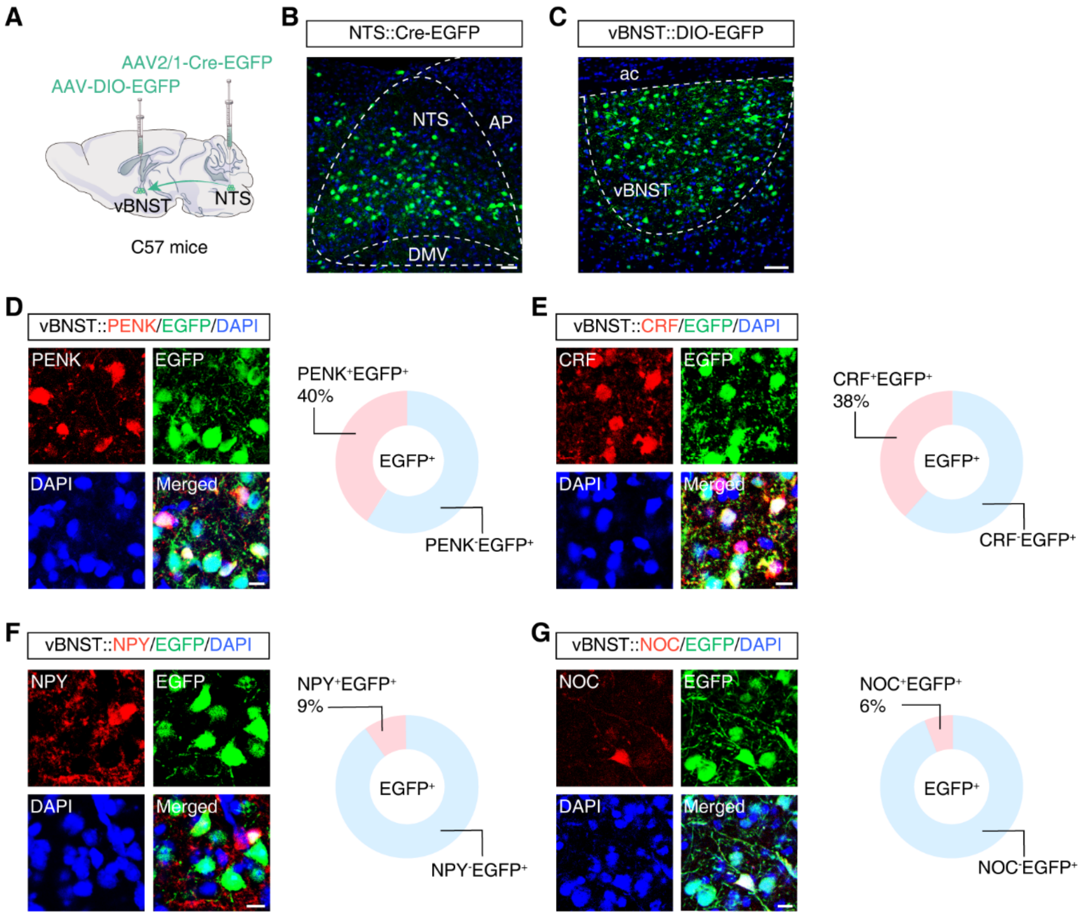

Fig. S17. vBNST cell type populations receiving projections from the NTS.

(*A*) Schematic for characterizing NTS-innervated vBNST neurons by viral injection.

(*B* and *C*) Representative images of EGFP signal at the (*B*) AAV2/1-Cre-EGFP injection site in the NTS and (*C*) AAV-DIO-EGFP injection site in the vBNST. Scale bars, 50 μm.

(*D* to *G*) Representative images and quantitative analysis of EGFP signals colocalized with signals for PENK (~40%), CRF (~38%), NPY (~9%), NOC (~6%) antibodies. Scale bars, 20 μm.

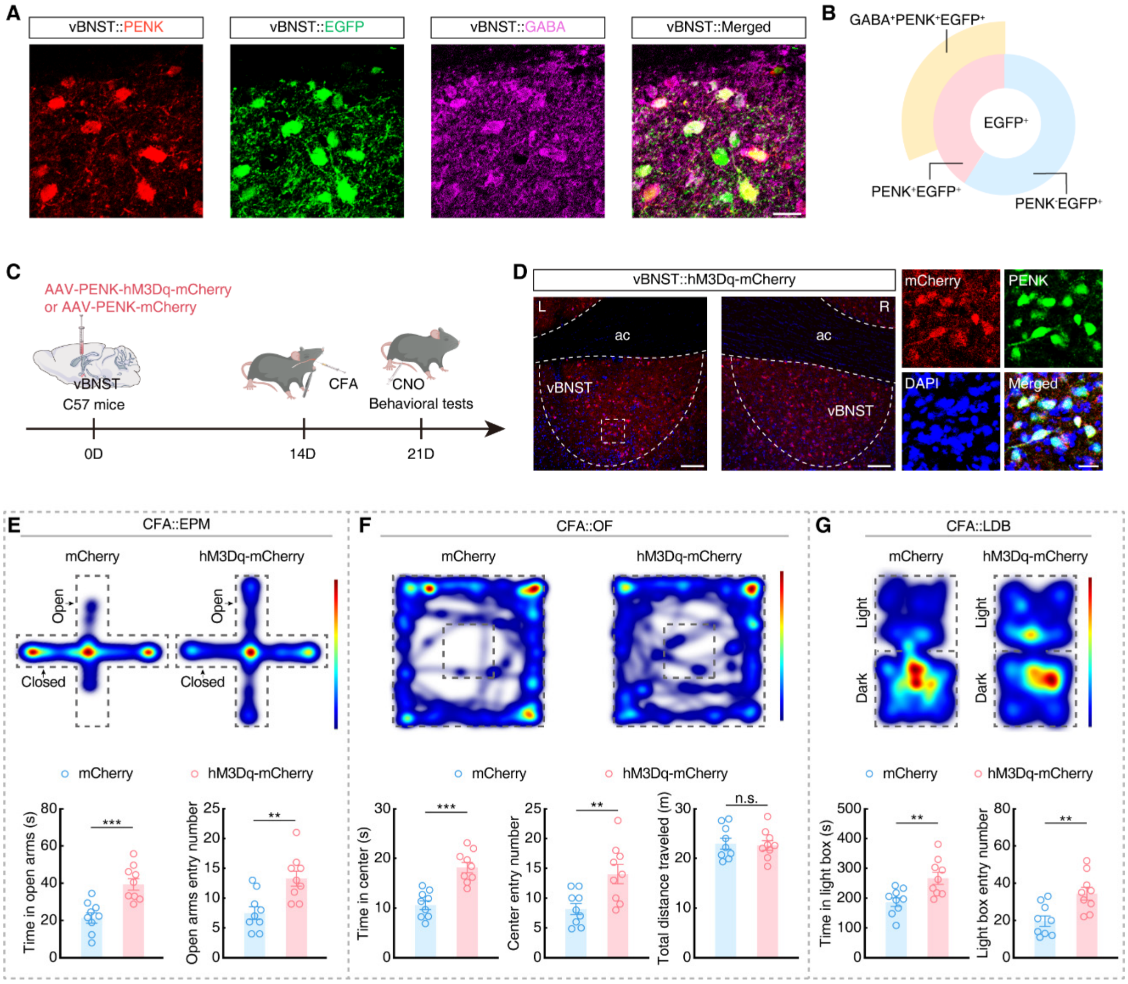

Fig. S18. Activation of vBNST^{ENK} neurons alleviates pharyngeal inflammation-induced anxiety-like behaviors.

(*A* and *B*) Representative images (*A*) and quantitative analysis (*B*) of ~78% GABA antibody signal colocalized with PENK$^+$EGFP$^+$ neurons. Scale bar, 20 μm.

(*C*) Schematic of viral injection and chemogenetic activation of vBNST^{ENK} neurons.

(*D*) Representative images showing the expression of hM3Dq-mCherry in the vBNST (left) and colocalized with PENK antibody (right). Scale bars, 100 μm or 20 μm. L: left; R: right.

(*E* to *G*) Representative heatmaps of the travel trajectory and summarized data for the EPM (*E*), OF (*F*) and LDB (*G*) tests with mCherry and hM3Dq-mCherry CFA mice (after injection of CNO, $n = 9$ mice per group; *E*, left, t_{16} = 4.297, P = 0.0006; right, t_{16} = 3. 482, P = 0.0031; *F*, left, t_{16} = 5.497, P < 0.0001; middle, t_{16} = 3.163, P = 0.0060; right, t_{16} = 0.2506, P = 0.8053; *G*, left, t_{16} = 3.194, P = 0.0056; right, t_{16} = 3.340, P = 0.0042).

Significance was assessed by two-tailed unpaired Student's *t* tests in (*E*, *F* and *G*). All data are presented as the mean ± SEM. **P < 0.01, ***P < 0.001, n.s., not significant.

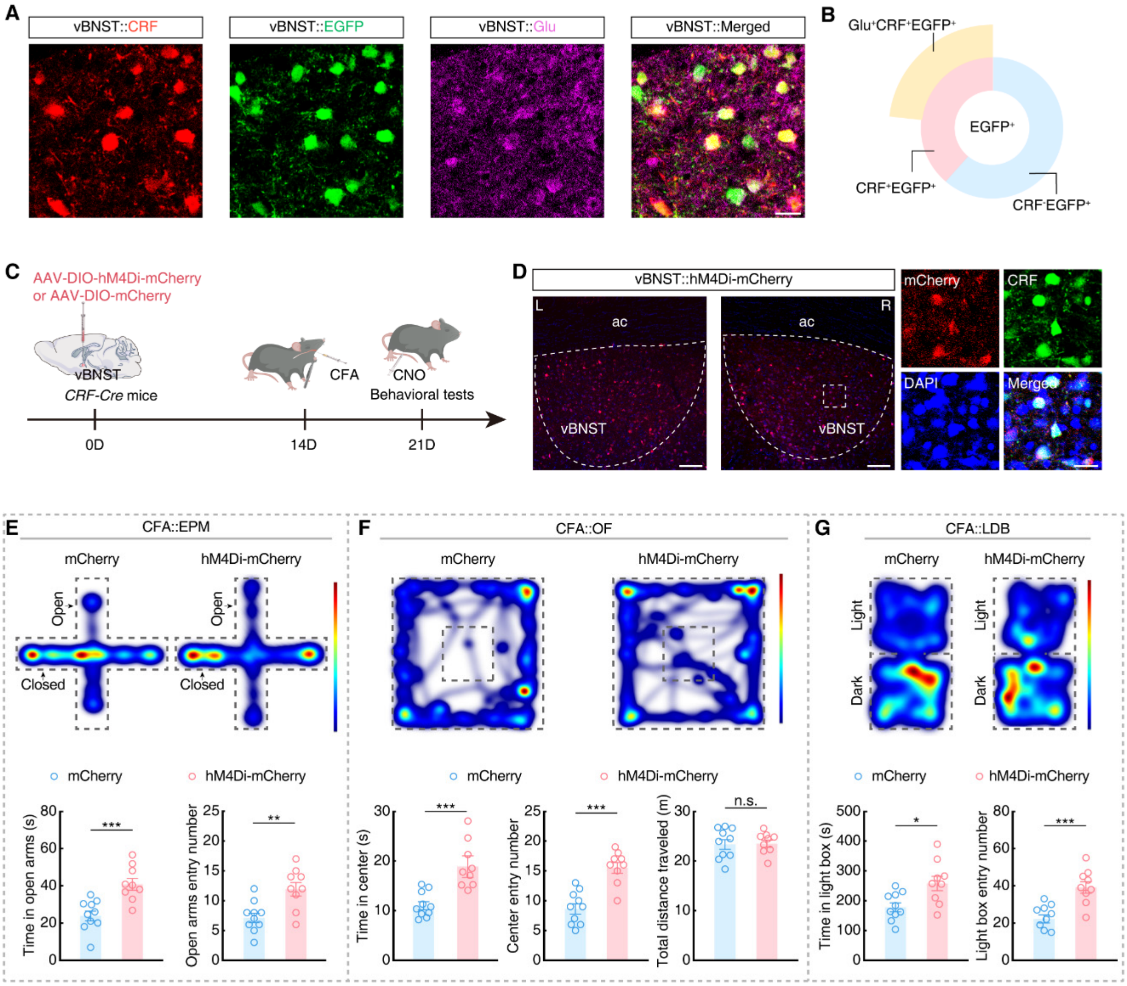

Fig. S19. Inhibition of vBNST^{CRF} neurons alleviates pharyngeal inflammation-induced anxiety-like behaviors.

(*A* and *B*) Representative images (*A*) and quantitative analysis (*B*) of ~60% glutamate antibody colocalized with CRF$^+$EGFP$^+$ neurons. Scale bar, 20 μm.

(*C*) Schematic of viral injection and chemogenetic inhibition design.

(*D*) Representative images showing the expression of hM4Di-mCherry in the vBNST (left) and colocalized with CRF antibody (right). Scale bars, 100 μm or 20 μm. L: left; R: right.

(*E* to *G*) Representative heatmaps of the travel trajectory and summarized data for the EPM (*E*), OF (*F*) and LDB (*G*) tests with mCherry and hM4Di-mCherry CFA mice (after injection of CNO; mCherry, n = 10 mice; hM4Di-mCherry, n = 9 mice; *E*, left, t_{17} = 4.183, P = 0.0006; right, t_{17} = 3.490, P = 0.0028; *F*, left , t_{17} = 4.609, P = 0.0003; middle, t_{17} = 5.455, P < 0.0001; right, t_{17} = 0.1026, P = 0.9195; *G*, left, t_{17} = 2.858, P = 0.0109; right, t_{17} = 4.519, P = 0.0003).

Significance was assessed by two-tailed unpaired Student's t tests in (*E*, *F* and *G*). All data are presented as the mean ± SEM. *P < 0.05, **P < 0.01, ***P < 0.001, n.s., not significant.

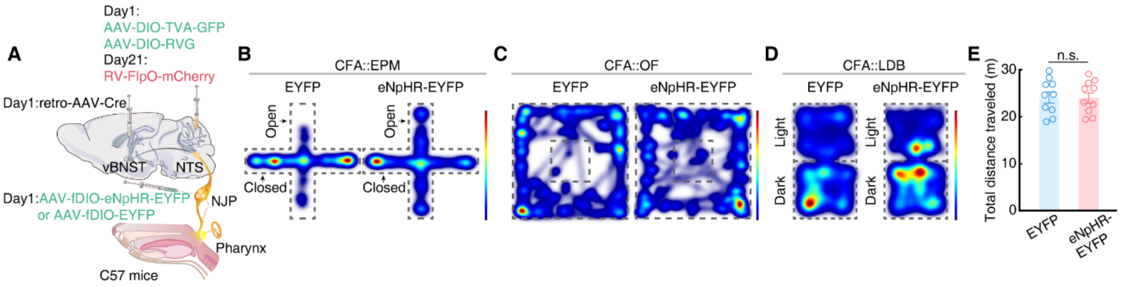

Fig. S20. Inhibition of pharynx→NJP→NTS^{NE}→vBNST^{Glu} circuit relieves pharyngeal inflammation-induced anxiety-like behaviors.

(*A*) Schematic of the viral injection.

(*B* to *D*) Representative heatmaps of the travel trajectory of EYFP and eNpHR-EYFP CFA mice in the EPM (*G*), OF(*H*) and LDB (*I*) tests.

(*E*) Summarized data for the total distance traveled by EYFP and eNpHR-EYFP CFA mice in the OF test (n = 10 mice per group; t_{18} = 0.05863, P = 0.9539).

Significance was assessed by two-tailed unpaired Student's *t* test in (*E*). All data are presented as the mean ± SEM. n.s., not significant.

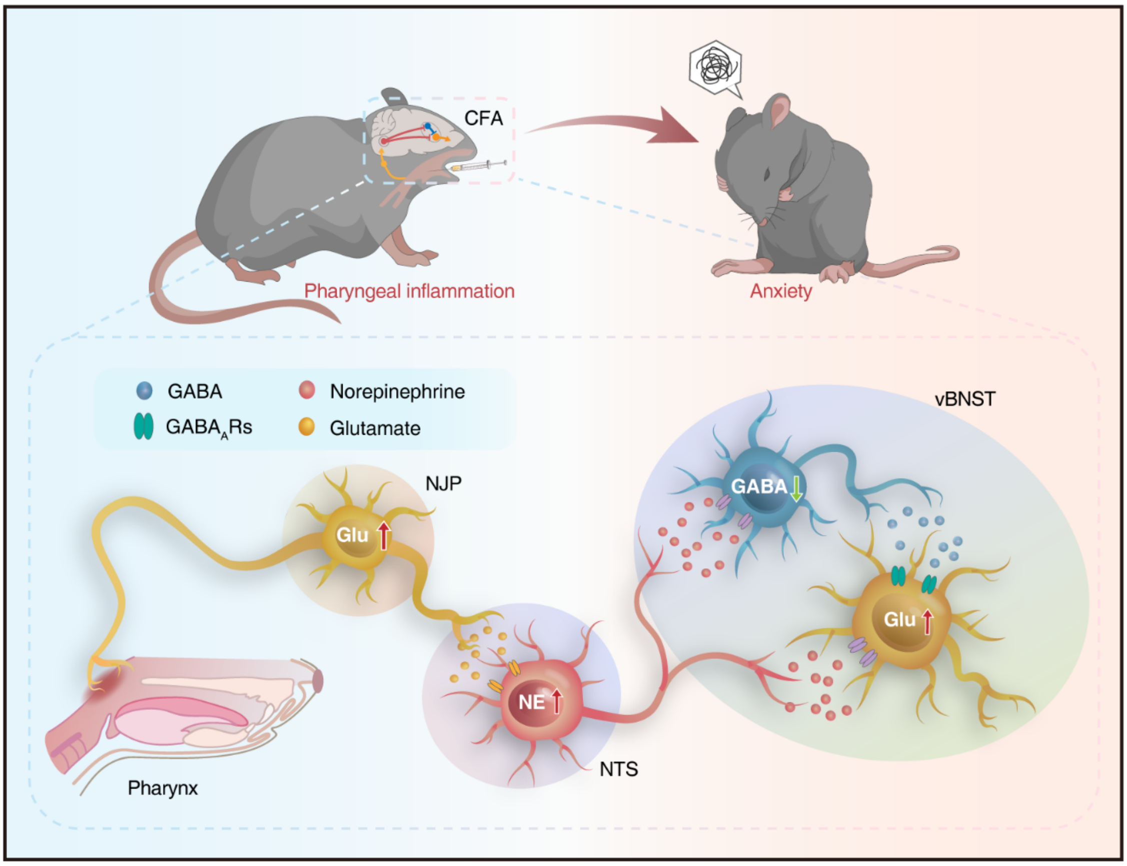

Fig. S21. A pharynx-to-brain axis controls pharyngeal inflammation-induced anxiety.

At 7 days after CFA-induced pharyngeal inflammation in mice, norepinephrinergic neurons in the nucleus of the solitary tract (NTS^{NE}) are activated by increased inputs from the inflammatory pharynx via glossopharyngeal and vagal sensory neurons of the nodose/jugular/petrosal (NJP) superganglia. The NTS^{NE} neurons in turn project to the ventral bed nucleus of the stria terminalis (vBNST), leading to anxiety-like behaviors in the murine model with pharyngeal inflammation. Ultimately, this pharynx→NJP→NTS^{NE}→vBNST circuit mechanistically links pharyngeal inflammation with glossopharyngeal and vagal sensory nerves and the induction of anxiety-like behaviors in mice. NJP, the nodose/jugular/petrosal superganglia; NTS, the nucleus of the solitary tract; vBNST, the ventral bed nucleus of the stria terminalis; NE, norepinephrine; Glu, glutamate; GABA, γ-aminobutyric acid; CFA, Complete Freund's Adjuvant.

Table S1. Survey questions and response options.

Survey questions/ description	Response
**Section 1: General Information**	
1. Age	1 = < 18 years<br>2 = 18-60 years<br>3 = > 60 years
2. Gender	1 = Male<br>2 = Female
3. Residence	1 = Urban<br>2 = Rural
4. Smoke	1 = Yes<br>2 = No
5. Drink	1 = Yes<br>2 = No
6. History of somatic disorders	1 = Yes<br>2 = No
7. History of psychiatric illness	1 = Yes<br>2 = No
8. History of head and neck surgery	1 = Yes<br>2 = No
9. History of anatomical abnormalities	1 = Yes<br>2 = No
10. History of severe systemic disease	1 = Yes<br>2 = No
11. History of cancer	1 = Yes<br>2 = No
**Section 2: Symptoms**	
12. Sore throat or pharyngeal discomfort	1 = Yes<br>2 = No
13. Sensation of a foreign body in the pharynx	1 = Yes<br>2 = No
14. Dry pharynx	1 = Yes<br>2 = No
15. Throat clearing	1 = Yes<br>2 = No
16. Chronic cough	1 = Yes<br>2 = No
17. Excessive mucus production	1 = Yes<br>2 = No
18. Difficulty swallowing	1 = Yes<br>2 = No
19. Duration of pharyngeal discomfort	1 = < 1 month<br>2 = 1-3 months<br>3 = > 3 months
**Section 3: Physical Examination**	
20. Pharyngeal hyperemia	1 = Yes<br>2 = No
21. Mucopurulent secretions	1 = Yes<br>2 = No
22. Catarrhal secretions	1 = Yes<br>2 = No

23. Lymphoid follicles on posterior wall	1 = Yes<br>2 = No
24. Mucosal atrophy	1 = Yes<br>2 = No
Tonsillo-Pharyngitis Assessment (TPA)(1, 2)	
25. Oral temperature	0 = ≤ 98.6°F<br>1 = 98.7 – 98.9°F<br>2 = 99.0 – 99.9°F<br>3 = ≥ 100.0°F
26. Oropharyngeal color	0 = Normal/pink<br>1 = Slightly red<br>2 = Red<br>3 = Beefy red
27. Size of tonsils	0 = Normal/absent<br>1 = Slightly enlarged<br>2 = Moderately enlarged<br>3 = Much enlarged
28. Number of oropharyngeal enanthems (vesicles, petechiae, or exudates)	0 = None<br>1 = Few<br>2 = Several<br>3 = Many
29. Largest size of anterior cervical lymph nodes	0 = Normal<br>1 = Slightly enlarged<br>2 = Moderately enlarged<br>3 = Much enlarged
30. Number of anterior cervical lymph nodes	0 = Normal<br>1 = Slightly enlarged<br>2 = Moderately enlarged<br>3 = Greatly enlarged
31. Maximum tenderness of some anterior cervical lymph nodes	0 = Not tender<br>1 = Slightly tender<br>2 = Moderately tender<br>3 = Very tender
**Section 4: Anxiety**	
Self-rating anxiety scale (SAS)(3, 4)	
32. I feel more nervous and anxious than usual.	1 = None or a little of the time<br>2 = Some of the time<br>3 = Good part of time<br>4 = Most or all the time
33. I feel afraid for no reason at all.	1 = None or a little of the time<br>2 = Some of the time<br>3 = Good part of time<br>4 = Most or all the time
34. I get upset easily or feel panicky.	1 = None or a little of the time<br>2 = Some of the time<br>3 = Good part of time<br>4 = Most or all the time
35. I feel like I'm falling apart and going to pieces.	1 = None or a little of the time<br>2 = Some of the time<br>3 = Good part of time<br>4 = Most or all the time
36. I feel that everything is all right and nothing bad will happen.	1 = None or a little of the time<br>2 = Some of the time<br>3 = Good part of time<br>4 = Most or all the time

37. My arms and legs shake and tremble.	1 = None or a little of the time<br>2 = Some of the time<br>3 = Good part of time<br>4 = Most or all the time
38. I am bothered by headaches, neck, and back pain.	1 = None or a little of the time<br>2 = Some of the time<br>3 = Good part of time<br>4 = Most or all the time
39. I feel weak and get tired easily.	1 = None or a little of the time<br>2 = Some of the time<br>3 = Good part of time<br>4 = Most or all the time
40. I feel calm and can sit still easily.	1 = None or a little of the time<br>2 = Some of the time<br>3 = Good part of time<br>4 = Most or all the time
41. I can feel my heart beating fast.	1 = None or a little of the time<br>2 = Some of the time<br>3 = Good part of time<br>4 = Most or all the time
42. I am bothered by dizzy spells.	1 = None or a little of the time<br>2 = Some of the time<br>3 = Good part of time<br>4 = Most or all the time
43. I have fainting spells or feel like it.	1 = None or a little of the time<br>2 = Some of the time<br>3 = Good part of time<br>4 = Most or all the time
44. I can breathe in and out easily.	1 = None or a little of the time<br>2 = Some of the time<br>3 = Good part of time<br>4 = Most or all the time
45. I get numbness and tingling in my fingers and toes.	1 = None or a little of the time<br>2 = Some of the time<br>3 = Good part of time<br>4 = Most or all the time
46. I am bothered by stomach aches or indigestion.	1 = None or a little of the time<br>2 = Some of the time<br>3 = Good part of time<br>4 = Most or all the time
47. I have to empty my bladder often.	1 = None or a little of the time<br>2 = Some of the time<br>3 = Good part of time<br>4 = Most or all the time
48. My hands are usually dry and warm.	1 = None or a little of the time<br>2 = Some of the time<br>3 = Good part of time<br>4 = Most or all the time
49. My face gets hot and blushes.	1 = None or a little of the time<br>2 = Some of the time<br>3 = Good part of time<br>4 = Most or all the time
50. I fall asleep easily and get a good night's rest.	1 = None or a little of the time<br>2 = Some of the time<br>3 = Good part of time

	4 = Most or all the time
51. I have nightmares.	1 = None or a little of the time<br>2 = Some of the time<br>3 = Good part of time<br>4 = Most or all the time

Movie S1 (separate file). *In vivo* microendoscopic calcium imaging of NTS^{NE} neurons in saline (left) and CFA (right) *DBH-Cre* mice, which NTS infusion of AAV-DIO-GCaMP6m.

Movie S2 (separate file). *In vivo* microendoscopic calcium imaging of $vBNST^{Glu}$ neurons in saline (left) and CFA (right) *VgluT2-Cre* mice, which vBNST infusion of AAV-DIO-GCaMP6m.

SI References

1. B. P. Schachtel, *et al.*, Utility and sensitivity of the sore throat pain model: results of a randomized controlled trial on the COX-2 selective inhibitor valdecoxib. *J Clin Pharmacol* **47**, 860–870 (2007).
2. B. Schachtel, *et al.*, Utility of the sore throat pain model in a multiple-dose assessment of the acute analgesic flurbiprofen: a randomized controlled study. *Trials* **15**, 263 (2014).
3. W. W. Zung, A rating instrument for anxiety disorders. *Psychosomatics* **12**, 371–379 (1971).
4. W. W. Zung, Prevalence of clinically significant anxiety in a family practice setting. *Am J Psychiatry* **143**, 1471–1472 (1986).
5. H. Sakai, M. Misawa, Effect of sodium azulene sulfonate on capsaicin-induced pharyngitis in rats. *Basic Clin Pharmacol Toxicol* **96**, 54–59 (2005).
6. G. L. Viswanatha, *et al.*, Novel experimental model of non-infectious pharyngitis in rats. *Journal of Pharmacological and Toxicological Methods* **69**, 189–195 (2014).
7. W. Zhou, *et al.*, Sound induces analgesia through corticothalamic circuits. *Science* **377**, 198–204 (2022).
8. Y.-H. Chen, *et al.*, Anti-inflammatory effect of Ganluyin, a Chinese classic prescription, in chronic pharyngitis rat model. *BMC Complement Med Ther* **20**, 265 (2020).
9. Y. Y, T. Y, H. M, N. T, Sensory innervation of the pharynx and larynx. *The American journal of medicine* **108 Suppl 4a** (2000).
10. S. L. Prescott, B. D. Umans, E. K. Williams, R. D. Brust, S. D. Liberles, An Airway Protection Program Revealed by Sweeping Genetic Control of Vagal Afferents. *Cell* **181**, 574-589.e14 (2020).
11. B. Zingg, *et al.*, AAV-Mediated Anterograde Transsynaptic Tagging: Mapping Corticocollicular Input-Defined Neural Pathways for Defense Behaviors. *Neuron* **93**, 33–47 (2017).
12. J.-J. Meng, *et al.*, Light modulates glucose metabolism by a retina-hypothalamus-brown adipose tissue axis. *Cell* **186**, 398-412.e17 (2023).
13. C. M. Contreras, *et al.*, Myristic Acid Produces Anxiolytic-Like Effects in Wistar Rats in the Elevated Plus Maze. *Biomed Res Int* **2014**, 492141 (2014).
14. R. Gong, S. Xu, A. Hermundstad, Y. Yu, S. M. Sternson, Hindbrain Double-Negative Feedback Mediates Palatability-Guided Food and Water Consumption. *Cell* **182**, 1589-1605.e22 (2020).
